# Mendelian randomization analysis to investigate the gut microbiome in oral and oropharyngeal cancer

**DOI:** 10.3389/fcimb.2023.1210807

**Published:** 2024-01-04

**Authors:** Qihe Zhang, Huanhuan Wang, Yuan Tian, Jinjie Li, Ying Xin, Xin Jiang

**Affiliations:** ^1^ Jilin Provincial Key Laboratory of Radiation Oncology & Therapy, The First Hospital of Jilin University, and Key Laboratory of Pathobiology, Ministry of Education, Jilin University, Changchun, China; ^2^ Department of Radiation Oncology, The First Hospital of Jilin University, Changchun, China; ^3^ NHC Key Laboratory of Radiobiology, School of Public Health, Jilin University, Changchun, China; ^4^ Key Laboratory of Pathobiology, Ministry of Education, Jilin University, Changchun, China

**Keywords:** gut microbiome, oral cancer, oropharyngeal cancer, Mendelian randomization, meta-analysis

## Abstract

**Background:**

Evidence supports an observational association between the gut microbiome and susceptibility to extraintestinal cancers, but the causal relationship of this association remains unclear.

**Methods:**

To identify the specific causal gut microbiota of oral and oropharyngeal cancer, we performed two-sample Mendelian randomization (MR) analysis of gut microbiota on oral and oropharyngeal cancer using a fixed-effects inverse-variance-weighted model. Gut microbiota across five different taxonomical levels from the MiBioGen genome-wide association study (GWAS) were used as exposures. Oral cancer, oropharyngeal cancer and a combination of the two cancers defined from three separate data sources were used as the outcomes. Odds ratios (ORs) and 95% confidence intervals (CIs) for disease per standard deviation (SD) higher abundance of microbiome.

**Results & Conclusions:**

There was little evidence for a causal effect of gut microbiota on oral and oropharyngeal cancer when using a genome-wide p-value threshold for selecting instruments. Secondary analyses using a more lenient p-value threshold indicated that there were 90 causal relationships between 58 different microbial features but that sensitivity analyses suggested that these were possibly affected by violations of MR assumptions and were not consistent across MR methodologies or data sources and therefore are also to unlikely reflect causation. These findings provide new insights into gut microbiota-mediated oral and oropharyngeal cancers and warrant further investigation.

## Introduction

Head and neck cancer is the sixth most common cancer worldwide (Warnakulasuriya, 2009), with oral and oropharyngeal cancers being the most common subtypes. Tobacco and alcohol consumption (Hashibe et al., 2009), human papillomavirus (HPV) infection (Ang et al., 2010), and specific sexual behaviors (Heck et al., 2010) have been recognized as oral and oropharyngeal cancer risk factors. Recently, there has been growing recognition of links between cancer and the microbiome; in particular, cancer-associated biomarkers have been observed in the gut microbiome (Cullin et al., 2021). The gut microbiota is a collection of bacterial species present in the intestinal tract. The roles of gut microbes in tumors can be divided into local and distal roles (Matson et al., 2021). In addition to the prominent role that specific gut microbes possess in local carcinogenesis, gut microbes can also alter the host’s overall immune system, leading to cancer (Castellarin et al., 2012; Amieva and Peek, 2016). There is a natural anatomical barrier between intestinal microorganisms and the intestinal epithelium, primarily composed of goblet cells that secrete intestinal mucus (Kim and Ho, 2010) and Paneth cells that produce antimicrobial peptides (Salzman et al., 2007). Therefore, the contact between intestinal microorganisms and the immune system is limited. However, specific microorganisms affect the integrity of the gut barrier. When this integrity is disrupted, an increased number of carcinogens circulate through the impaired gut barrier (Rajagopala et al., 2017); furthermore, inflammation or immunosuppression are induced, playing an indirect role in promoting cancer (Yu and Schwabe, 2017). An example illustrating this distal role is that the gut microbiota can promote hepatocellular carcinoma and pancreatic cancer growth/progression/invasion and metastasis, which contain no known microbiome, by elevating cancer-promoting inflammatory microbial-associated molecular patterns such as lipopolysaccharides (Dapito et al., 2012; Ochi et al., 2012).

The administration of probiotics to regulate the immune system is a potential antitumor strategy (Vétizou et al., 2015). Gut microbes can modulate immunity by regulating the primary and secondary lymphoid organs of the intestinal epithelial barrier, thereby affecting the tumor microenvironment (Gopalakrishnan et al., 2018). An association between gut microbes and intestinal tumor susceptibility has been previously reported (Yachida et al., 2019). Gut microbiota have been shown to affect the body’s immune response by regulating immune cell function, affecting inflammatory response, regulating immune tolerance (Zhou et al., 2021), and producing metabolites (Zhang et al., 2019). However, the causal relationship between the gut microbiota and parenteral tumors, especially oropharyngeal and oral cancers, remains unclear.

Mendelian randomization (MR) is a statistical method used to assess the causal relationship between exposure and outcome, based on instrumental variables (genetic variants) which can be viewed as a natural analog of randomized controlled trials (RCTs). In contrast to traditional gold-standard RCTs, participants are assigned according to their genotype, reducing the impact of reverse causality and confounding factors such as ethical and socioeconomic factors. Therefore, we aimed to investigate whether the gut microbiota is causally related to oral and oropharyngeal cancers using two-sample MR which uses summary-level data, typically from genome-wide association studies (GWASs).

## Methods

### Study design and data sources

**Figure 1:**
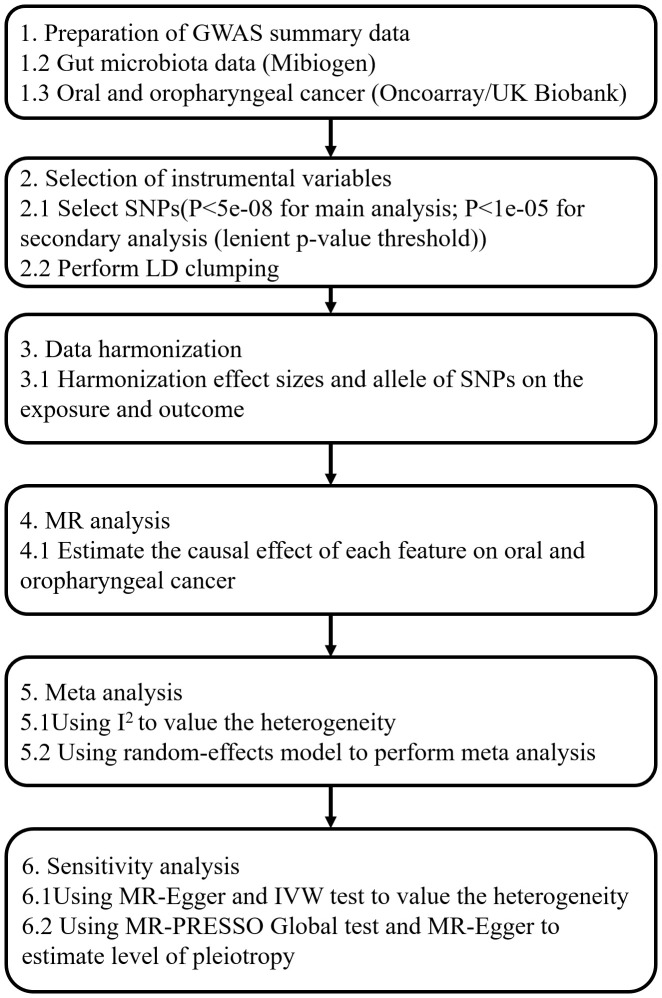
The whole workflow of MR analysis.

Our MR analysis used gut microbiota features as the exposure data and oral and oropharyngeal cancer data as the outcome (**Figure 1**). The original investigation was conducted after obtaining ethical approval for each study included in the MR analysis. Genome-wide association study (GWAS) data for the gut microbiota were obtained from the MiBioGen study (Kurilshikov et al., 2021) and the oral and oropharyngeal cancer data were obtained from the MRC IEU OpenGWAS data resource (Elsworth et al., 2020). The original oral and oropharyngeal cancers data were released from OncoArray Oral Cavity and Oropharyngeal Cancer Consortium (Lesseur et al., 2016) and UK Biobank (Burrows et al., 2021). The MiBioGen consortium includes 24 cohorts and totals 18340 participants. In each cohort, the gut microbiome is analyzed through 16S rRNA sequencing using Illumina platforms (MiSeq or HiSeq), while the participants undergo genotyping using whole-genome SNP arrays. All outcomes were based on European ancestry. Each outcome data source we used contained three outcomes: oral cancer, oropharyngeal cancer, and their combination. The OncoArray study comprised a cohort of 6,034 cases and 6,585 controls. The number of cases and controls in North America were 2549 (42.2% of total cases) and 2522 (38.3% of total controls), respectively. In Europe, 2499 cases (41.4% of total cases) and 2928 controls (44.5% of total controls) were reported. For Hispanic or Latin American, OncoArray included 986 cases (16.3%) and 1135 controls (17.2%). Because our MR Study was limited to European populations and gut microbial exposures were not available with respect to Hispanic or Latin American populations, we therefore excluded outcomes if they were Hispanic or Latin American from the study which were restricted to only those of North American and European ancestry. Given the ethnic diversity, Lesseur et al. evaluated associations within continent (Europe, North and South America) using multivariate unconditional logistic regressions under a log-additive genetic model adjusted for age, sex and regional eigenvectors. Genotyping of 13,107 individuals was conducted using Illumina platforms across 12 epidemiological studies. After rigorous quality control measures, a total of 6,034 cases and 6,585 cancer-free controls were included in the final analysis, 4839 cases and 5257 controls of which were of European decent and therefore were used in this MR analysis. Whole-genome imputation was performed using the Haplotype Reference Consortium panel, resulting in approximately 7 million high-quality imputed variants. The UK Biobank began in 2006 by recruiting approximately 500,000 participants between the ages of 38 and 73. Participants completed a series of questionnaires that provided detailed personal and lifestyle information. In addition, participants provided biospecimens, including blood, urine, and saliva, which were subsequently sequenced for genome sequencing and genotyping using the Illumina sequencing platform in the UK Biobank. Details of outcomes are provided in **Table 1**. The number of total cases of oral and oropharyngeal cancer combined and controls of the UK Biobank were 839 and 372016, respectively.

**Table 1:** Outcome GWAS samples used in this study.

GWAS ID	Trait	Consortium	Sample size	Number of SNPs	Population	ncase	ncontrol	PMID/DOI
ieu-b-89	Oral cavity and pharyngeal cancer	OncoArray oral cavity and oropharyngeal cancer	5,425	7,514,278	European (Geographic region: Europe)	2,497	2,928	27749845
ieu-b-94	Oral cavity cancer	OncoArray oral cavity and oropharyngeal cancer	4,151	7,510,833	European (Geographic region: Europe)	1,223	2,928	27749845
ieu-b-96	Oropharyngeal cancer	OncoArray oral cavity and oropharyngeal cancer	4,018	7,508,444	European (Geographic region: Europe)	1,090	2,928	27749845
ieu-b-90	Oral cavity and pharyngeal cancer	OncoArray oral cavity and oropharyngeal cancer	4,671	7,510,261	European (Geographic region: North America)	2,342	2,329	27749845
ieu-b-93	Oral cavity cancer	OncoArray oral cavity and oropharyngeal cancer	3,464	7,506,142	European (Geographic region: North America)	1,135	2,329	27749845
ieu-b-97	Oropharyngeal cancer	OncoArray oral cavity and oropharyngeal cancer	3,448	7,506,485	European (Geographic region: North America)	1,119	2,329	27749845
ieu-b-4962	Oral and oropharyngeal cancer	UK Biobank	372,855	9,185,233	European	839	372,016	10.5523/bris.aed0u12w0ede20olb0m77p4b9
ieu-b-4961	Oral cavity cancer	UK Biobank	372,373	7,723,107	European	357	372,016	10.5523/bris.aed0u12w0ede20olb0m77p4b9
ieu-b-4968	Oropharyngeal cancer	UK Biobank	372,510	8,283,869	European	494	372,016	10.5523/bris.aed0u12w0ede20olb0m77p4b9

GWAS, Genome-wide association study; ncase, Number of case; ncontrol, Number of control; PMID, ID of study in Pubmed; DOI, Digital Object Identifier.

### Selection of instrumental variables

We initially extracted instrumental variables using a genome-wide association study p-value threshold of 5e-08. Subsequently, Kurilshikov et al. identified a total of 30 quantitative SNP-feature associations through mbQTL analysis (Kurilshikov et al., 2021). After conducting the main Mendelian randomization (MR) analysis for these exposures, we further performed a secondary analysis using a more lenient threshold criteria, specifically, we extracted five levels of GWAS summary data of exposure (phylum, class, order, family, and genus) derived from MiBioGen, where the number of features were 9, 16, 20, 35, and 131 respectively. Single-nucleotide polymorphisms (SNPs) smaller than the p-value threshold within the locus-wide range (1 × 10^-5^) were selected as instrumental variables according to a previous study (Sanna et al., 2019). One MR principle is that there is no linkage disequilibrium (LD) between the included instrumental variables, as a strong LD may lead to biased results, for strong LD may bias the results by genetic confounding or overestimating the association between the genetic variants used and the exposure and outcome. In this study, the SNPs were clumped within each phenotype, clumping processing (R^2^< 0.1, clumping distance = 500 kb) (Ni et al., 2021) was performed to evaluated LD among the SNPs used to instrument each phenotype separately, where SNPs in LD were removed and the strongest (i.e., that with the smallest p-value) was retained for MR analyses.

### Assumptions

The two-sample MR study relies on three critical assumptions to minimize bias. First, the genetic instruments used were robustly associated with exposure. Second, there is no confounding factor between the instrument and the outcome. Third, instrumental variables affected the outcomes only through exposure, meaning that there was no horizontal pleiotropy effect between instrumental variables and outcomes.

### Statistical methods and multiple testing correction

When combining summarized data on genetic associations, it is necessary to ensure that genetic associations are expressed per additional copy of the same allele. If a genetic variant is a biallelic single nucleotide polymorphism (SNP) with alleles A and G on the positive strand, then the corresponding base pairs on the negative strand will be T and C. In this case, one dataset may report the association per additional copy of the A allele, and another per additional copy of the T allele – but the same comparison is being made. Allele and strand information can be double-checked by comparing allele frequency information – if the allele frequencies are similar for the A and T alleles, this means that this is a strand mismatch. For palindromic variants – if the alleles were A and T (or C and G), then the same alleles would appear on both the positive and negative strands. In such a case, if the allele frequency is close to 50%, it may be necessary to drop the variant from the analysis if it is not possible to verify that the alleles have been correctly orientated. While this is a conservative recommendation, allele alignment problems have led to incorrect results in MR analyses. Therefore, palindromic SNPs with intermediate (i.e., 0.42%) frequencies were discarded. Analytical methods used included the Wald ratio and inverse variance weighted (IVW) estimators (Burgess et al., 2013). The Wald ratio estimates the causal effect of the exposure on the outcome using a single instrumental variable (IV). When horizontal pleiotropy is absent, IVW results are considered robust. In general, the Wald ratio was used for the analysis with only one SNP and the IVW method was used for the analysis with multiple SNPs The IVW has the strictest assumptions; therefore, this method was considered the main analytical method, to which results from all other methods were compared. Effect estimates and 95% confidence intervals (CIs) reflect the odds ratios (ORs) for disease per standard deviation (SD) higher abundance of microbiome.

In our study, we chose not to apply multiple testing correction in both primary and secondary analyses due to the complex interactions and correlations among microbial features. Although multiple testing correction is common in many studies, we believe that it might be overly stringent in this context, potentially obscuring true causal relationships. This decision is grounded in the complexity of the microbial ecosystem, where microbes often influence each other. Given these interactions, applying multiple testing correction to each microbial feature may be overly conservative and might obscure some signals that have genuine causal relationships.

### Colocalization analysis

Colocalization analysis is commonly employed to ascertain whether two phenotypes are driven by the same causal variant within a particular genomic region, thereby bolstering evidence for the association between the two phenotypes. For colocalisation analyses, we extracted SNPs that fell within a 500 kb window upstream and downstream of the SNP used as an instrument for the microbial feature from both the microbiome and oral cancer GWAS datasets. To be more specific, we acquired GWAS summary data from the MRC IEU OpenGWAS data resource. Using the SNPs extracted during the primary MR analysis, we queried their chromosomal number and position within the PheWAS - IEU OpenGWAS project (mrcieu.ac.uk). With this positional information, we adjusted the numerical value by either adding or subtracting 500,000 to establish a positional range spanning 500kb upstream and downstream. With the chromosome number and positional range, we then conducted a filter operation on the GWAS summary data previously downloaded, resulting in the final selection of SNPs located within a 500kb radius both up and downstream of the instrument. The number of extracted SNPs is shown in **Supplementary Table 2**.

Specifically, we used the SNP positions that satisfy the threshold criteria and fall within a 500 kb window upstream and downstream as candidate SNPs for extraction in both exposure and outcome traits for colocalization analysis. Colocalization analysis involves five mutually exclusive model assumptions, namely: H0: no association exists between all SNP loci within a genomic region and both the exposure and outcome; H1/H2: significant association exists between the exposure/outcome, respectively, and SNP loci within a genomic region; H3: association exists between both exposure and outcome and SNP loci within a genomic region, driven by distinct causal variants; H4: association exists between both exposure and outcome and SNP loci within a genomic region, driven by the same causal variant. During the colocalization analysis, posterior probabilities (PP.H0-PP.H4) are generated for each of these models. The sum of the posterior probabilities for the five models equals 1. A higher posterior probability for a specific model indicates a higher likelihood of that model assumption being valid given the data. We considered the model assumption to be valid when the posterior probability of that model was greater than 0.80. Priors are as follows: p1: prior probability a SNP is associated with microbiome trait, default is 1e-4; p2: prior probability a SNP is associated with outcome, default is 1e-4; p12: prior probability a SNP is associated with both exposure and outcome, default is 1e-5.

Our colocalization analysis was based on any result obtained from the primary MR analysis that suggested a causal effect of an exposure the OncoArray outcomes. First, we identified SNPs within a 500kb range for both exposure and outcome, and then merged the data based on their rsID, aligning effect alleles. We calculated the variance (VAR) after splitting and cleaning the data by removing missing values, specifying phenotype types: binary phenotype as “cc” (for outcomes) and continuous phenotype as “quant” (for exposure). We conducted colocalization analysis using the “coloc” package and generated locus zoom plots using the “locuscompare” package.

### Assessment of assumptions

We approximated the variance explained in each gut microbial trait by the respective associated genetic instruments using the following Equation 1 (Shim et al., 2015; Papadimitriou et al., 2020):


(1) $$R^{2}=\frac{(2\times EAF\times (1-EAF)\times beta^{2})}{\left(2\times EAF\times \left(1-EAF\right)\times beta^{2}\right)+\left(2\times EAF\times \left(1-EAF\right)\times N\times SE^{2}\right)}$$

In this context, EAF represents the frequency of the effect allele, beta signifies the estimated genetic impact on exposure, N stands for the sample size of the GWAS concerning the association between the SNP and exposure, and SE represents the standard error of the genetic effect. Since EAF was not available for raw data on gut microbes, we inquired the allele frequency (GRCh37) of each SNP through the 1000 genome project (https://www.internationalgenome.org/1000-genomes-browsers/index.html). The power of our MR analyses was assessed using the online calculator mRnd (https://shiny.cnsgenomics.com/mRnd/).

### Sensitivity analyses

Maximum likelihood (Pierce and Burgess, 2013), MR-Egger (Bowden et al., 2015), weighted median (Bowden et al., 2016), weighted mode, and MR-PRESSO (Verbanck et al., 2018) were used to infer potential causality. The Maximum likelihood (ML) method bears resemblance to IVW, provided there is no presence of heterogeneity and horizontal pleiotropy. If these assumptions hold true, the outcomes will remain unbiased, and the standard errors will be smaller compared to IVW (Yavorska and Burgess, 2017). We used the MR-Egger intercept term to assess pleiotropy. In the presence of horizontal pleiotropy, the MR-Egger effect estimate is a more accurate representation of the unbiased causal effect. If this intercept term was close to zero, then the MR-Egger regression model would be similar to the IVW model. However, if the intercept term was different from zero, it indicates that there may have been horizontal pleiotropy among these IVs. The weighted median can provide consistent estimates of causal effects, providing<50% of SNPs are invalid. We assessed the robustness of the results using sensitivity analysis and tested heterogeneity using Cochran’s Q-test in the IVW test and MR-Egger regression. We conducted MR-PRESSO tests to correct for horizontal pleiotropic effect by removing the IV outliers. MR-Egger regression was used to estimate the effect of pleiotropy; it produced a more robust pleiotropy-corrected causal estimate under the assumptions of no measurement error and instrument strength independent of direct effects (Burgess and Thompson, 2017). Leave-one-out analysis was used to determine whether the original causal effect was driven by a single SNP. We also used the MR Steiger test to examine evidence for directionality in the relationship (i.e., that the exposure caused the outcome rather than the reverse) by comparing the variance explained in the exposure and outcome by the exposure-related IV (Hemani et al., 2017). Furthermore, we assessed instrument strength using the F statistic (Burgess and Thompson, 2011), calculated using the Equation 2:


(2) $$\text{F}=\frac{R^{2}(N-k-1)}{k\left(1-R^{2}\right)}$$

where R^2^ represents the variance of exposure explained by the selected SNPs, N is the sample size, and k represents the number of instrumental variables. If F< 10, indicating a higher likelihood of weak instrument bias, the association between instrumental variables and exposure was considered weak.

### Meta-analyses

We had three outcome datasets: OncoArray (including North American and European individuals treated as separate data sources) and the UK Biobank. The meta-analysis involved meta-analysing the IVW-specific estimates of the microbial features on the same outcomes across different datasets. The meta-analysis employed both a random-effects model and a fixed-effects model, and was conducted using the meta-package in R. Heterogeneity between studies was assessed using I^2^ and associated p-value. We first determined whether the direction of the effect size for each microbial feature was consistent across the different methods within each data source. If the direction was consistent across methods, we conducted a meta-analysis. If the effect size direction for a particular microbial feature differed among methods for any of the three data sources, then meta-analysis was not performed for that microbial feature.

### Software and pre-registration

Analysis was performed using R software (version 4.0.2; R Foundation for Statistical Computing, Vienna, Austria), with the R packages “TwoSampleMR” (version 0.5.6) (Hemani et al., 2018) and “MRPRESSO” (Verbanck et al., 2018). We followed STROBE-MR (guidelines for strengthening the reporting of observational studies in epidemiological studies using MR to report our results (Skrivankova et al., 2021).

## Results

### Primary MR analysis

We employed a genome-wide association study p-value threshold of 5e-08 to extract instrumental variables. Our results are consistent with those of Kurilshikov et al., as a total of 30 SNPs associated with 27 microbial phenotypes were extracted (Kurilshikov et al., 2021). Among these, genus *Intestinibacter*, genus *RuminococcaceaeUCG009*, and genus *CandidatusSoleaferrea* yielded one extracted SNP each (rs10805326, rs8009993, and rs830151, respectively), yet no corresponding SNP was found in the outcome. Conversely, for genus *Peptococcus*, genus *Enteror-habdus*, genus *Ruminococcus1*, and genus *Faecalibacterium*, the sole SNPs (rs75754569, rs11098863, rs10769159, and rs12320842, respectively) were discarded for being palindromic with intermediate allele frequencies.

We proceeded to perform MR analysis on the remaining 20 gut microbial features. Wald ratio results indicated that order *Gastranaerophilales*, family *Gastranaerophilales*, and genus *Gastranaerophilales* exhibited carcinogenic effects on oral and oropharyngeal cancer in the North American population from OncoArray with odds ratios (ORs) per standard deviation (SD) higher abundance of 2.610 [1.124-6.061] (oral and oropharyngeal cancer) and 3.609 [1.232-10.568] (oropharyngeal cancer). However, these effects were inconsistent across multiple data sources, as the causal effect directions of order *Gastranaerophilales*, family *Gastranaerophilales*, and genus *Gastranaerophilales* varied across different data sources (**Supplementary Table 1**). Subsequently, we conducted colocalization analysis on these 30 SNPs, revealing that none of the PP.H4 values exceeded 0.80, providing limited evidence in support of the hypothesis that “exposure and outcome are associated with SNP loci within a genomic region, driven by the same causal variant.”(**Supplementary Table 2**) (**Supplementary Figure 1**).

### Secondary MR analysis

Subsequently, we conducted analyses using a more lenient p-value threshold. We extracted five levels of GWAS summary data on exposure derived from MiBioGen. After the removal of palindromic SNPs, clumping, and harmonization of the data, the number of SNPs associated with the microbial traits that were available in the outcome data ranged from 4 to 22; the microbial trait associated with the fewest SNPs was the genus *Hungatella*, with four SNPs extracted, and the microbial trait associated with the most SNPs was the class *Actinobacteria*, with 22 SNPs extracted (**Supplementary Table 3**). The R^2^ and F-statistic values for the exposures are summarized in **Supplementary Table 4**. The R^2^ values for all SNPs of exposure were greater than the corresponding R^2^ for the SNPs of the outcome. The F-statistics for all exposures were greater than 10, indicating little evidence for weak instrument bias, which demonstrated that all SNPs had sufficient validity.

A secondary MR analysis provided evidence for causal relationships between 90 (includes 58 microbiota signatures) and oral cancers across the three data sources. There were 13, 15, and 17 microbiota features that caused a change in risk of oral cancer, oropharyngeal cancer, and combined oral and oropharyngeal cancer in the European population, respectively (**Supplementary Figures 2-13**), and nine, three, and eight microbiota features that caused a change in risk of oral cancer, oropharyngeal cancer, and combined oral and oropharyngeal cancer in the North American population, respectively (**Supplementary Figures 14-25**). The number of features that possessed causal relationships with oral cancer, oropharyngeal cancer, and combined oral and oropharyngeal cancer in the UK Biobank data were 10, 5, and 10, respectively (**Supplementary Figures 26-37**) (**Supplementary Table 3**).

**Figure 2:**
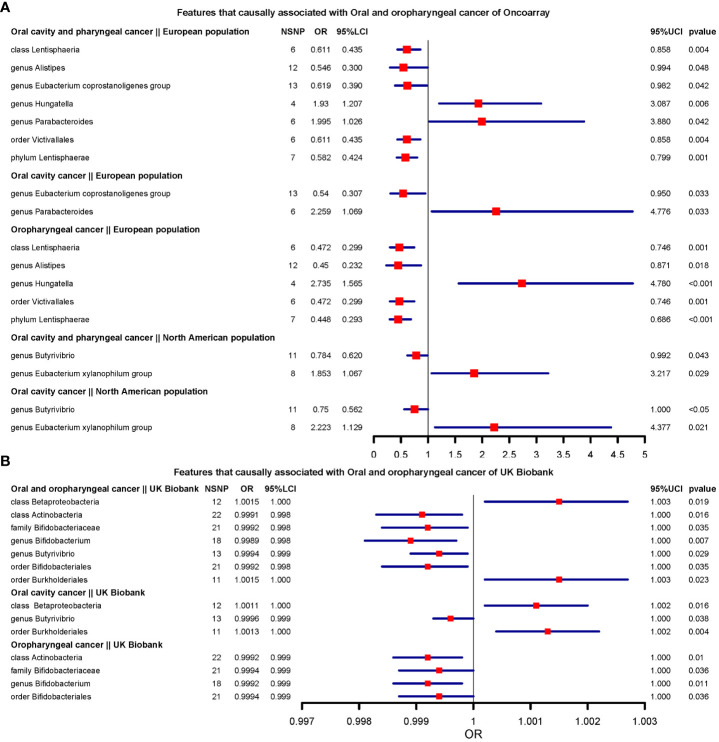
Features that causally associated with oral and oropharyngeal cancer of OncoArray and UK Biobank.**(A)** Forest plot of features that causally associated with Oral and oropharyngeal cancer of OncoArray; **(B)** Forest plot of features that causally associated with Oral and oropharyngeal cancer of UK Biobank.

We present the results of microbiota signatures with causal effects on oral or oropharyngeal cancer in combined oral and oropharyngeal cancer data, for these estimates were consistent in terms of direction of effect in all methods. For the European region, genus *Hungatella* (OR=2.735, [1.565;4.78] (Oropharyngeal cancer)), and *Parabacteroides* were risk factors, whereas the genus *Alistipes* and *Eubacterium coprostanoligenes* group, phylum *Lentisphaerae* (OR=0.448, [0.292;0.686] (Oropharyngeal cancer)), class *Lentisphaeria*, and order *Victivallales* were protective factors (**Figure 2A**).

In North American populations, one protective factor (genus *Butyrivibrio*) (OR=0.784, [0.619;0.991] (Oral cavity and oropharyngeal cancer)), and one risk factor (genus *Eubacterium xylanophilum group*) (OR=2.223, [1.129;4.376] (Oral cavity cancer)) were causally associated with combined oral and oropharyngeal cancers and oral or oropharyngeal cancer (**Figure 2A**).

In the UK Biobank data, there were seven microbial characteristics were associated with outcomes, among which class *Betaproteobacteria*, order *Burkholderiales*(OR=1.001, [1.000;1.002] (Oral cavity cancer)) were risk factors, whilst class *Actinobacteria*, family *Bifidobacteriaceae*, genus *Bifidobacterium*(OR=0.998, [0.998;0.999](Oral and oropharyngeal cancer)) and *Butyrivibrio*, and order *Bifidobacteriales* were protective factors. (**Figure 2B**).

Unfortunately, none of the signatures were consistent across the three data sources. However, there were 7 signatures that were consistent across the two data sources, including class *Lentisphaeria* (European region and North American region of OncoArray), genus *Butyrivibrio* (North American region of OncoArray and UK Biobank), order *Bacillales* (European region of OncoArray and UK Biobank), order *Bifidobacteriales* (European region of OncoArray and UK Biobank), order *Burkholderiales* (North American region of OncoArray and UK Biobank), order *Victivallales* (European region and North American region of OncoArray), family *Bifidobacteriaceae* (European region of OncoArray and UK Biobank). We compared the direction of the resulting effect size for the 20 exposures in the main analysis with the direction of the effect size obtained after the use of the lenient threshold criteria. Among the 180 causal relationships examined, 113 exhibited consistent effect directions in the secondary MR analysis following lenient threshold criteria. Of the 20 phenotypes assessed in the main analysis, none had directionally consistent effect estimates across main and secondary analyses across all methods in 9 outcomes. Notably, genus *Tyzzerella3* and genus *Romboutsia* maintained consistent directions for 8 effect estimates after the secondary analysis. The direction of effect for Gastranaerophilales on oral cavity and pharyngeal cancer and oropharyngeal cancer (Both from North American region of OncoArray) was the same as the direction of effect for Gastranaerophilales in the secondary analysis across all methods (**Supplementary Table 1**).

### Sensitivity analyses

The results of Cochran’s Q test suggested that there was little evidence of heterogeneity, where all p-values were greater than 0.05. The horizontal pleiotropy between instrumental variables and outcomes was assessed using the MR-Egger regression. There was little evidence of horizontal pleiotropy was found (**Supplementary Table 4**). MR-PRESSO global test results showed the presence of outliers only in the genus *Veillonella* analysis of Oral and oropharyngeal cancer (UK Biobank). After excluding outliers, the MR-PRESSO results showed that there was no evidence for a causal effect of *Veillonella* on either cancer. Additionally, the leave-one-out analysis showed that none of the identified causal associations were driven by any single IV. (**Supplementary Figures 2–37**).

In our secondary MR Analysis, 18 causal relationships (17 microbial traits) were found to be inconsistent in the direction of causal effects among different methodologies (**Supplementary Table 3**), so these causal relationships were excluded from the subsequent meta-analysis. There were 72 causal relationships (50 microbial traits) in the same direction of causal effects across methodologies (**Supplementary Table 3**).

We performed the MR Steiger directionality test to determine whether there was evidence that the assumed direction of causality was correct. All Steiger test p-values were less than 0.05, suggesting that the assumed direction of effect from the gut microbiome to oral and oropharyngeal cancers was correct, except for the genus *Hungatella* on oropharyngeal cancer (Steiger *P*-value=0.32). However, the R^2^ of exposure SNPs was greater than that of the outcome, suggesting that all causal directions were correct.

### Meta-analysis

For class *Lentisphaeria*, genus *Butyrivibrio*, genus *Ruminococcus 2*, order *Victivallales*, and phylum *Lentisphaerae* consistently aligned with the MR methods, and therefore, estimates of the effect of these microbial features on oral and oropharyngeal cancer from all three data sources were included in the meta-analysis. Our results showed that there was limited evidence suggesting a causal effect of any microbial feature on oral and oropharyngeal cancer in the meta-analysis (**Supplementary Table 5**). Next, we conducted a meta-analysis for oral cancer outcomes, using the same criteria as before. Although genus *Eubacterium xylanophilum group*, genus *Parabacteroides*, and genus *Ruminococcus gauvreauii group* consistently aligned with the MR methods, the meta-analysis results provided little evidence for causal effects (**Supplementary Table 6**).

Only the estimates of genus Ruminococcaceae UCG010 were consistent in direction across all methods in individual data sources, therefore, the meta-analysis was conducted with this one microbial feature. Results showed little evidence for a causal effect of Ruminococcaceae UCG010 on oropharyngeal cancer (**Supplementary Table 7**).

## Discussion

In the current study, we performed MR analyses to assess causal effect of gut microbial signatures on oral and oropharyngeal cancer. Firstly, the main analysis is where instruments have been selected based on a genome-wide p-value threshold. Although the initial analysis found a causal association between genus *Gastranaerophilales* and oropharyngeal cancer, despite initial analyses suggesting a causal effect, colocalisation analyses implied that these results are unlikely reflective of causality. Although we lowered the criteria for the extraction of instrumental variables and obtained 58 microbiota features with possible causal associations in secondary MR analysis, after meta-analysis of the 9 microbial features across each data source was consistent among different methods, random effects model showed limited evidence of causal effects. In addition, the main results of the meta-analysis were derived from secondary MR with lenient threshold criteria, which is likely biased given the lack of robust associations between instruments and the exposure. Therefore, based on the results of our primary MR analysis and colocalization analysis, which is a requirement for causality, we believe that the current evidence does not yet support the causal association of gut microbiota characteristics with oral and oropharyngeal cancer.

Whilst we were unable to provide strong evidence of horizontal pleiotropy in these analyses, it is possible (and likely) that the instruments we used may still be pleiotropic. While our outcome data originated from a sample of European individuals, it’s crucial to note that the MiBioGen data exhibit mixed ancestry, introducing the possibility of violating the second MR assumption due to genetic confounding. To establish causality, addressing confounding becomes imperative, wherein a third variable influences both exposure and outcome, creating a noncausal association. Genetic confounding is a nuanced scenario wherein genetic factors serve as this influential third variable. The inherent complexity of gene inheritance in organisms poses a challenge, making it arduous to fully isolate or control for the specific effects of individual genes, rendering the adjustment for genetic confounding difficult.

We observed that original data for microbial features under the same taxonomic classification (class *Lentisphaeria*-order *Victivallales*) and (order *Bifidobacteriales*-family *Bifidobacteriaceae*) were entirely identical, yielding the same OR values. This convergence could be attributed, on one hand, to potential characteristic dominant species within the same taxonomic classification, resulting in data congruence between the upper and lower taxonomic levels. On the other hand, order *Bifidobacteriales* predominantly segregates into family *Bifidobacteriaceae* and family *Gardnerellaceae*, with the latter recognized for its presence in maintaining vaginal microbial equilibrium in female reproductive tracts. Given that our exposure data were sourced from gut microbiota, data congruence between order *Bifidobacteriales* and family *Bifidobacteriaceae* was plausible. We opted for data representation at relatively lower taxonomic levels and refrained from analyzing analogous microbial features as independent exposures. For instance, we selected order *Victivallales* from (class *Lentisphaeria* and order *Victivallales*) and family *Bifidobacteriaceae* from (order *Bifidobacteriales* and family *Bifidobacteriaceae*) to curtail redundancy.

Understanding the causal relationship between gut microbiota and oral and oropharyngeal cancers can help guide decisions on health management and disease prevention strategies. Because GWAS data for the oral microbiome are unavailable, and oral microbiota has a more diverse and dynamic bacterial community than gut microbiota (Maki et al., 2021). Therefore, we selected the largest currently published GWAS for the gut microbiome from the MiBioGen consortium. Our study validated the features of the gut microbiota associated with cancer susceptibility. We performed analyses both with the genome-wide p-value threshold and with a more lenient p-value threshold, as a secondary analysis. Whilst extracted instrumental variables that did not meet the traditional genome-wide p-value threshold may very likely lead to weak instrument bias and violate core MR assumptions, the use of a lenient p-value threshold allowed the application of pleiotropy-robust methods in sensitivity analyses. And for those results with different causal directions from different MR methods, only those results with the same causal direction from all methods were reported. We assessed weak instrument bias through F-statistics and found little evidence for weak instruments. However, even if the F-statistic is greater than 10, instrumental variables may still be ineffective, as they could exhibit a high degree of pleiotropy (wherein ineffective instruments can still yield a high F-statistic) (Wade and Hall, 2019). Instrumental variables might be associated with outcomes through various channels, leading to violations of MR assumptions if independent from the exposure. We cannot rule out this possibility in our analysis. In addition to the pleiotropy-robust methods, the MR Steiger directionality test was used to test for the causal direction of effect and, in almost all causes, it provided evidence that the assumed causal direction (i.e., from the exposure to outcome) was correct. The genus *Hungatella* failed to pass, that is, it did not provide enough evidence to support the direction of causality. Therefore, genus *Hungatella* cannot be considered to have a causal effect on oropharyngeal cancer.

Moreover, augmenting the study’s sample size holds the potential to enhance statistical power, thereby elevating the probability of identifying associations. Larger sample sizes contribute to more accurate estimates and diminish p-values. The observed disparity in the significance of the association between a gut microbiome feature, as indicated by Oncoarray versus UK Biobank, may stem from variations in sample size. Despite the notably extensive size of the UK Biobank dataset, the proportion of cases within the total sample remains relatively modest. This discrepancy could have compromised statistical power, conceivably masking authentic effect signals that might otherwise be discernible. In addition, the MR-Egger method is sensitive to horizontal pleiotropy, that is, genetic variation affects the outcome independent of the exposure. Although the p value of the MR-Egger intercept in our study was greater than 0.05, there was indeed an unbalanced pleiotropic effect in our secondary analysis.

In our secondary MR results, the genus *Intestinibacter*, *Ruminiclostridium5*, and the order *Rhodospirillales* exhibited causal effect estimates using the MR-Egger method that differed in direction from those obtained through other methods (IVW, Maximum Likelihood, Weighted Median, etc.), suggesting violations of MR assumptions. The difference in power limitations between the MR-Egger estimator and other MR methods stems from the fact that the former estimates two parameters – the causal effect and the degree of unbalanced pleiotropy – whereas other methods focus solely on estimating the singular parameter, the causal effect. While our sensitivity analysis did not reveal the presence of pleiotropy, the results for these specific microbial features suggest a potential underlying pleiotropy. Additionally, several other microbial features displayed varying causal effects across different data sources, such as the family *Prevotellaceae* and the orders *Bacillales* and *Burkholderiales*. The family *Prevotellaceae* demonstrated a risk factor for oral and oropharyngeal cancer in European populations while being protective in North American populations. We hypothesize that these variations in the impact of these microbial features on oral and oropharyngeal cancer between North American and European populations could stem from differences in dietary habits and lifestyles, or potentially environmental influences on microbial composition and functionality. Furthermore, the sample size and diversity of the microbial communities under study could affect the stability of results. The divergence in outcomes between different regions could possibly be attributed to factors such as higher microbial diversity. However, our study’s primary objective was to explore microbial features associated causally with the occurrence of oral and oropharyngeal cancer in European populations. At present, there are no reports on variations in the effects of regional microbial features on oral and oropharyngeal cancer. Therefore, our interpretation of potential reasons for the differing impacts can only be approached with caution.

In our study, we’ve chosen not to apply multiple testing correction in the primary and secondary analyses due to the complex interactions and correlations among microbial features. While multiple testing correction is common, we believe it might be overly strict in this context, potentially hiding true causal relationships. We acknowledge this as a limitation and recommend future research to explore these interactions, develop precise statistical methods, and conduct larger studies for validation.

Our study initially provided evidence that features of the gut microbiome may influence oral and oropharyngeal cancers. However, due to likely violations of core MR assumptions and heterogeneity across both different methodologies that test those assumptions and across data sources of the same outcomes, our results indicate that gut microbiota may not play an important role in the development of oral and oropharyngeal cancers. In conclusion, we comprehensively evaluated the potential causal relationship between the gut microbiota and oral and oropharyngeal cancers. This study provides new insights into the mechanisms of microbial-mediated oral and oropharyngeal cancer development.

## Data availability statement

Publicly available datasets were analyzed in this study. This data can be found here: https://gwas.mrcieu.ac.uk/.

## Author contributions

Conceptualization, YX and XJ. Methodology, QZ. Software, QZ. Validation, YX and JX. Formal analysis, HW. Investigation, YT. Resources, XJ. Data curation, JL. Writing-original draft preparation, QZ and HW. Visualization, XJ. Supervision, XJ. Project administration, XJ. Funding acquisition, XJ and YX. All authors contributed to the article and approved the submitted version.
