# Supplementary material for: Mendelian randomization analysis to investigate the gut microbiome in oral and oropharyngeal cancer
Source: Front Cell Infect Microbiol. 2024 Jan 4;13:1210807. doi: 10.3389/fcimb.2023.1210807 (PMC10794669; doi:10.3389/fcimb.2023.1210807)
Supplement: Supplementary file 8 [file Presentation_1.pptx]

## Slide 1
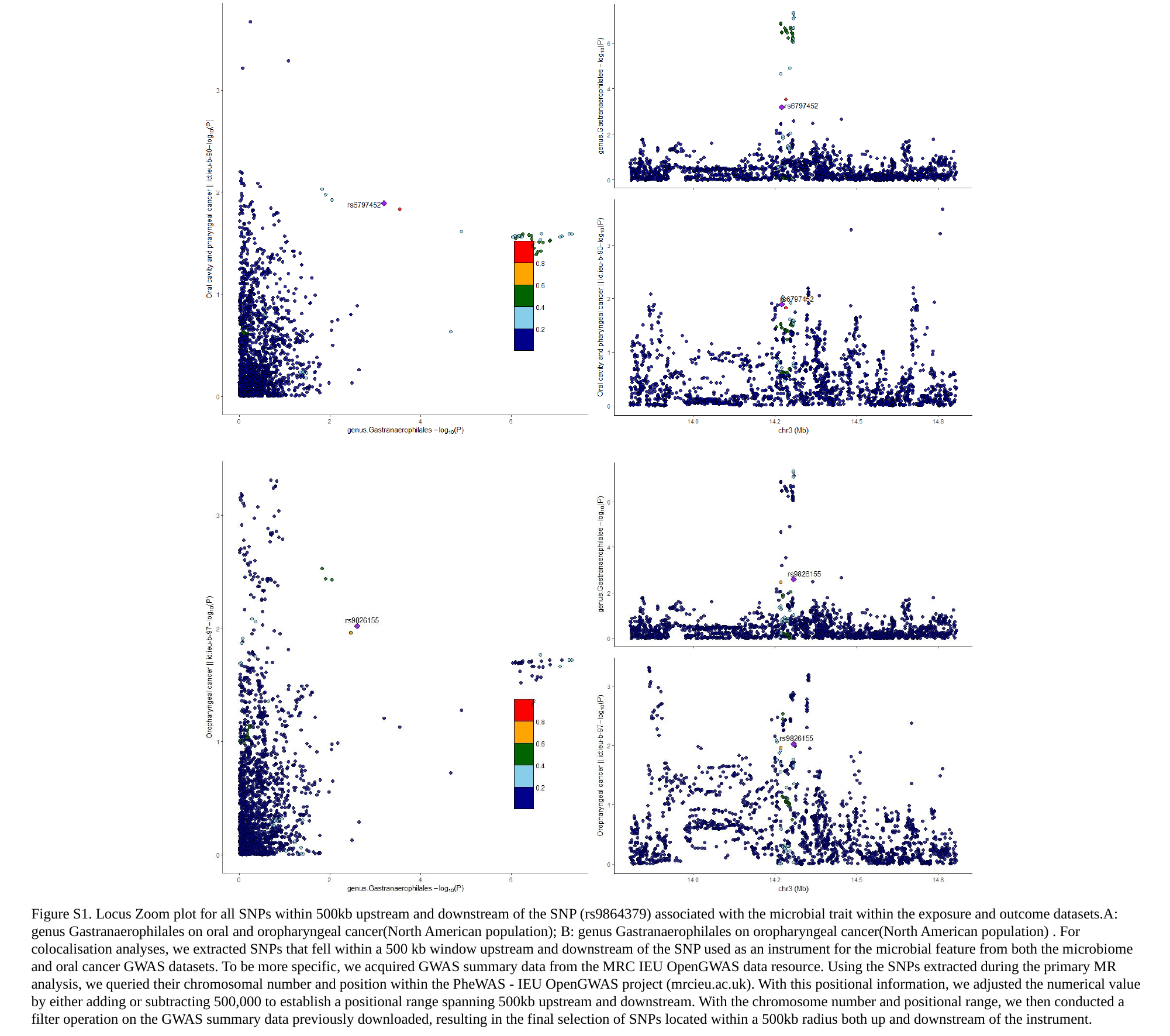

Figure S1. Locus Zoom plot for all SNPs within 500kb upstream and downstream of the SNP (rs9864379) associated with the microbial trait within the exposure and outcome datasets.A: genus Gastranaerophilales on oral and oropharyngeal cancer(North American population); B: genus Gastranaerophilales on oropharyngeal cancer(North American population) . For colocalisation analyses, we extracted SNPs that fell within a 500 kb window upstream and downstream of the SNP used as an instrument for the microbial feature from both the microbiome and oral cancer GWAS datasets. To be more specific, we acquired GWAS summary data from the MRC IEU OpenGWAS data resource. Using the SNPs extracted during the primary MR analysis, we queried their chromosomal number and position within the PheWAS - IEU OpenGWAS project (mrcieu.ac.uk). With this positional information, we adjusted the numerical value by either adding or subtracting 500,000 to establish a positional range spanning 500kb upstream and downstream. With the chromosome number and positional range, we then conducted a filter operation on the GWAS summary data previously downloaded, resulting in the final selection of SNPs located within a 500kb radius both up and downstream of the instrument.

## Slide 2
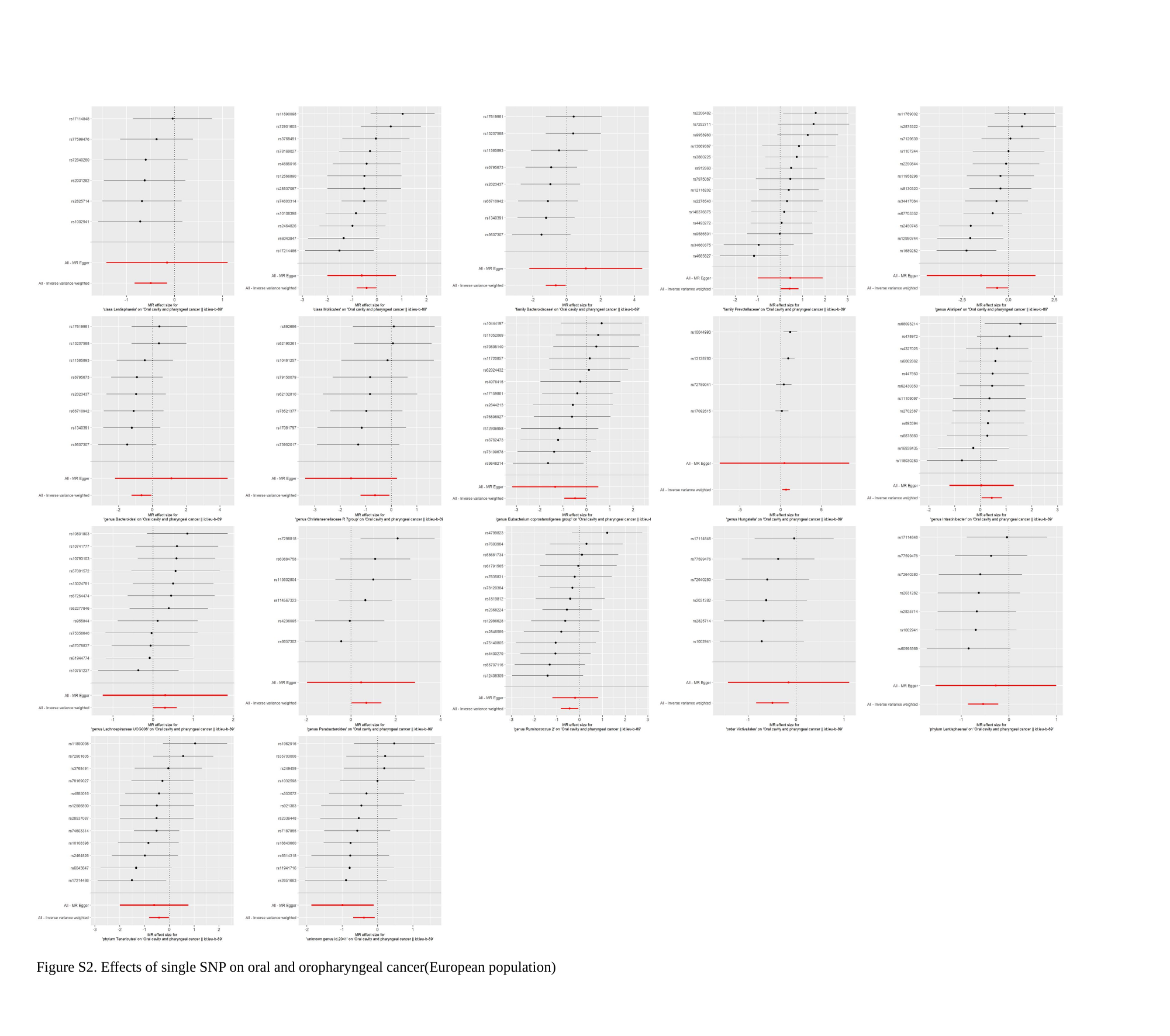

Figure S2. Effects of single SNP on oral and oropharyngeal cancer(European population)

## Slide 3
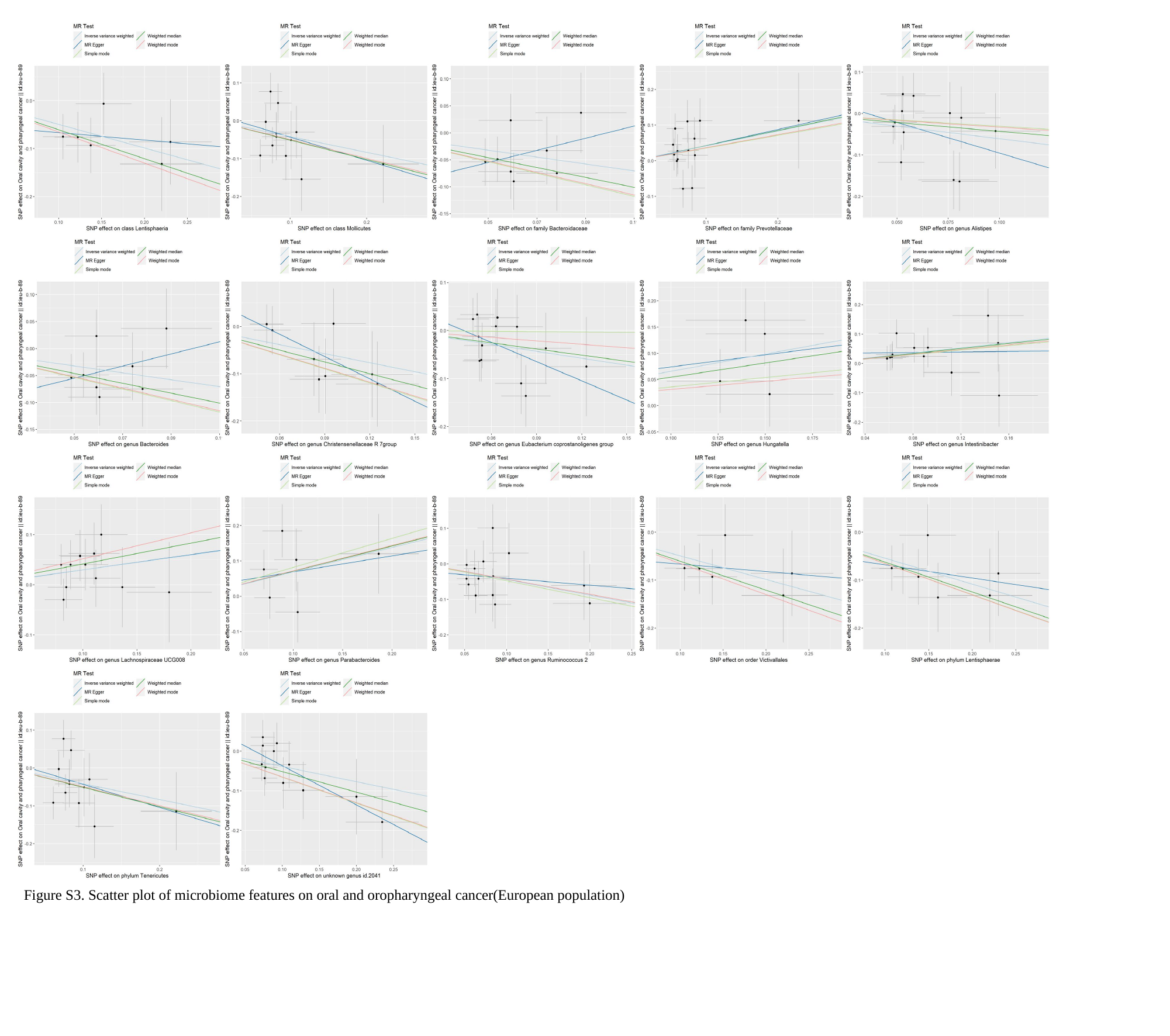

Figure S3. Scatter plot of microbiome features on oral and oropharyngeal cancer(European population)

## Slide 4
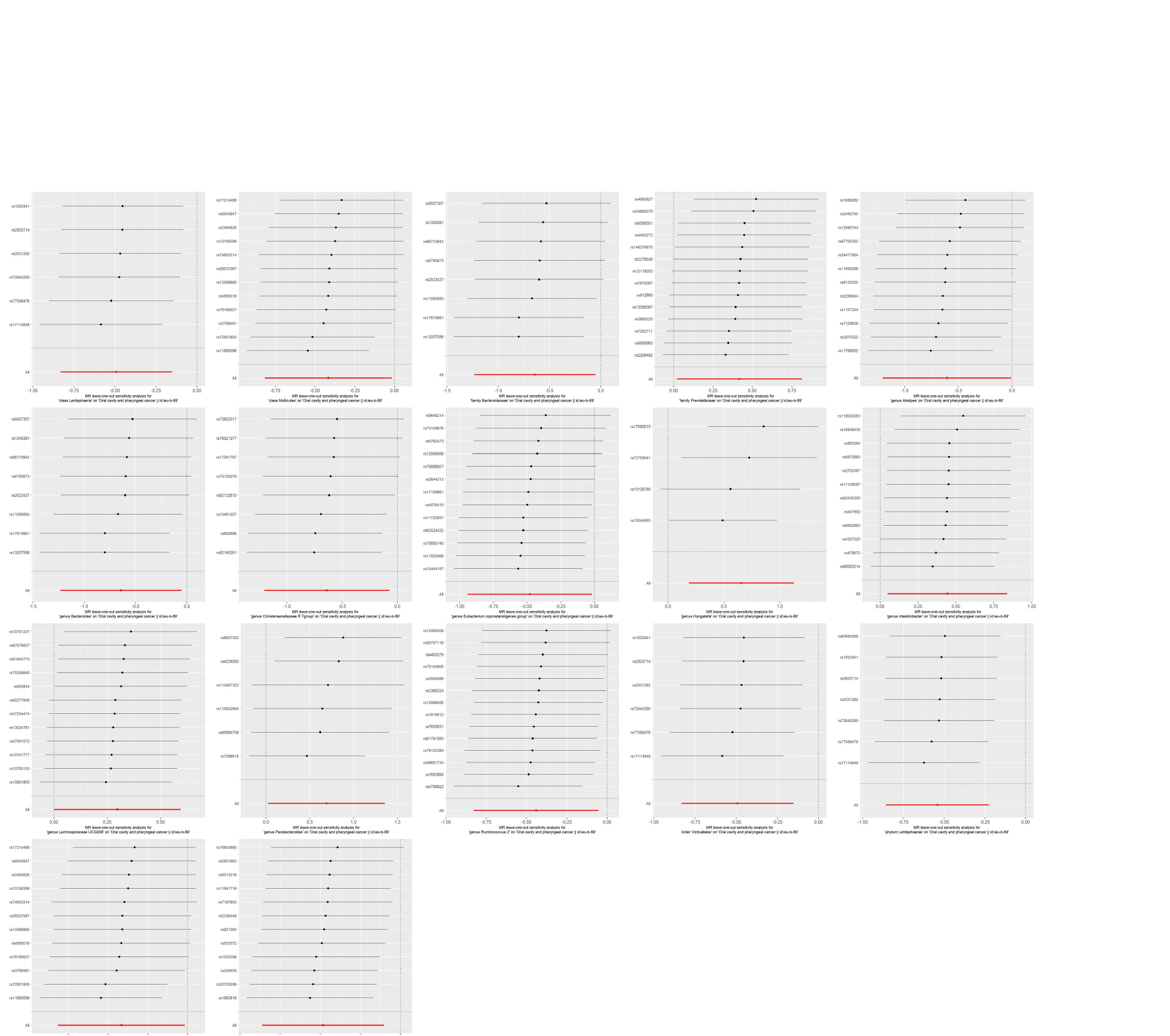

Figure S4. Leave-one-out plot of microbiome features on oral and oropharyngeal cancer(European population)

## Slide 5
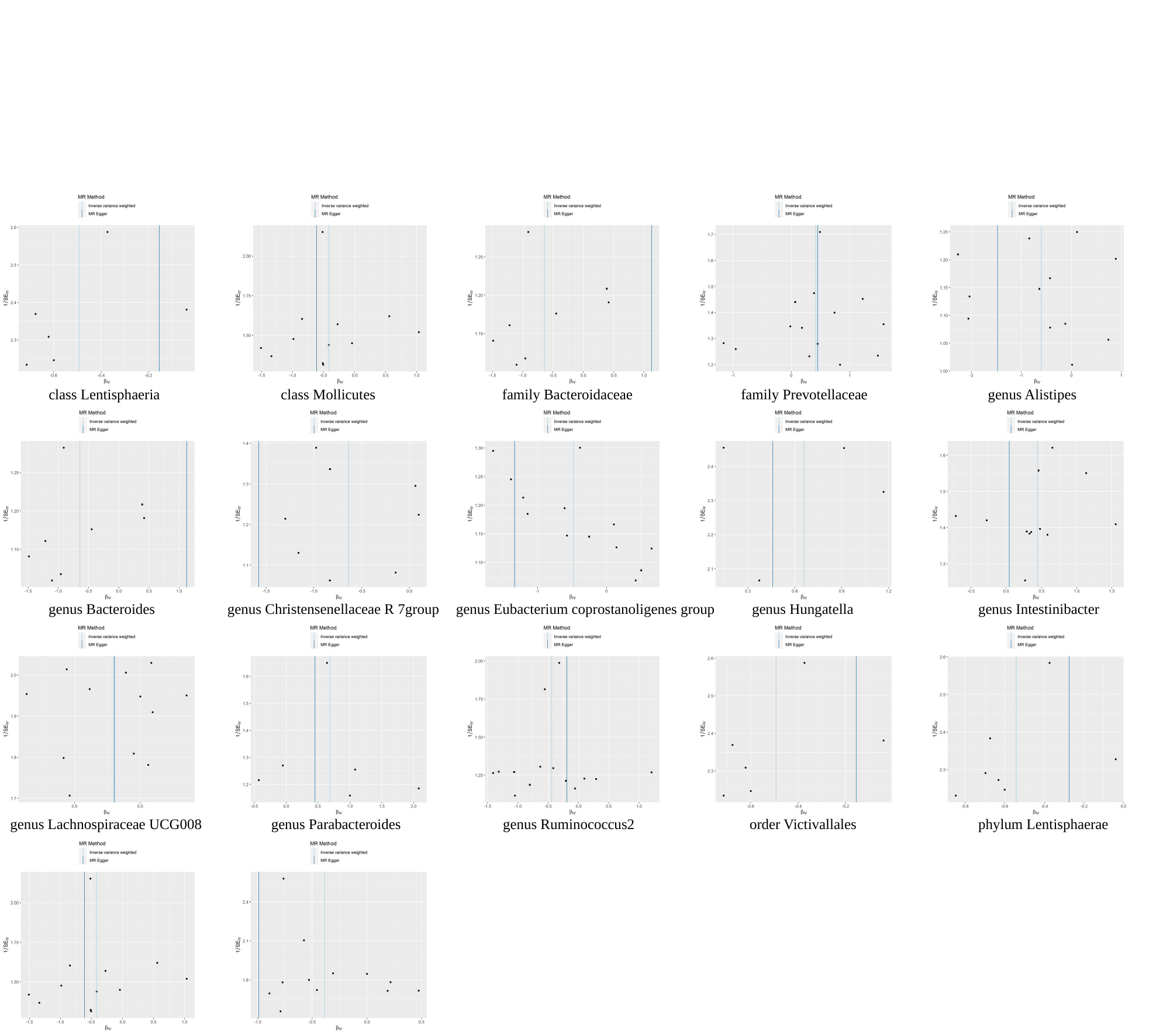

class Lentisphaeria
class Mollicutes
family Bacteroidaceae
family Prevotellaceae
genus Alistipes
genus Bacteroides
genus Christensenellaceae R 7group
genus Eubacterium coprostanoligenes group
genus Hungatella
genus Intestinibacter
genus Lachnospiraceae UCG008
genus Parabacteroides
genus Ruminococcus2
order Victivallales
phylum Lentisphaerae
phylum Tenericutes
unknown genus id.2041
Figure S5. Funnel plot of microbiome features on oral and oropharyngeal cancer(European population)

## Slide 6
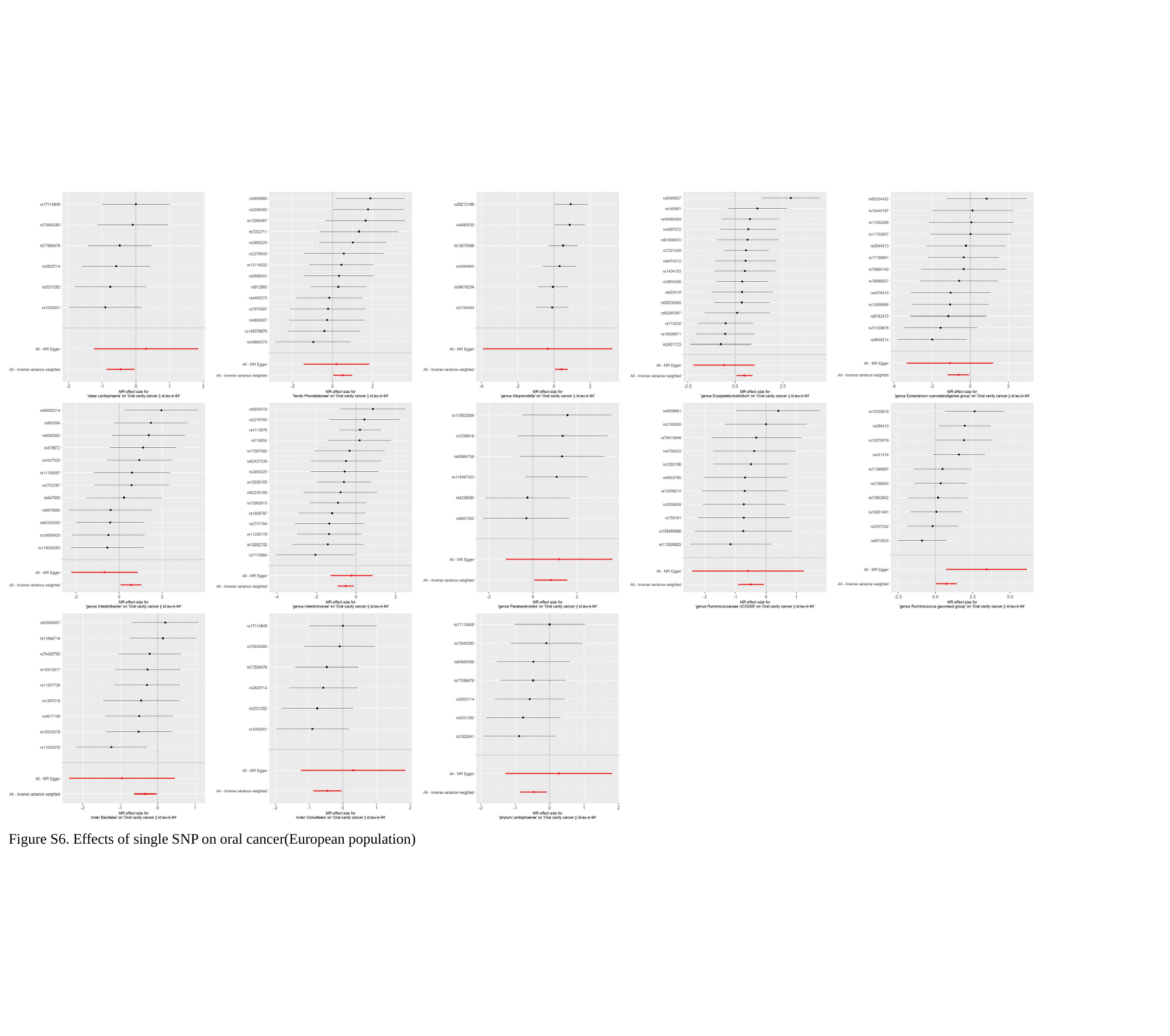

Figure S6. Effects of single SNP on oral cancer(European population)

## Slide 7
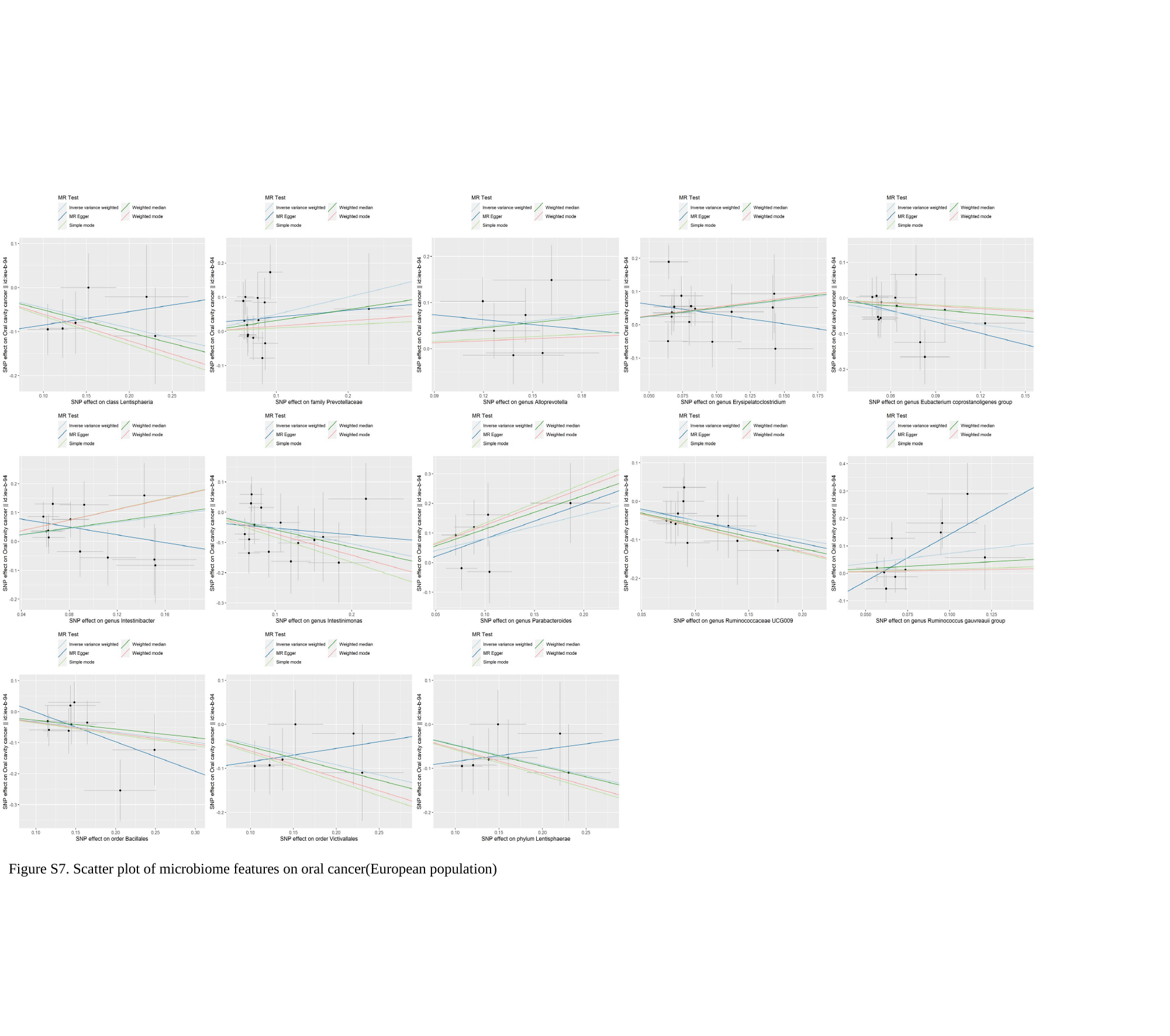

Figure S7. Scatter plot of microbiome features on oral cancer(European population)

## Slide 8
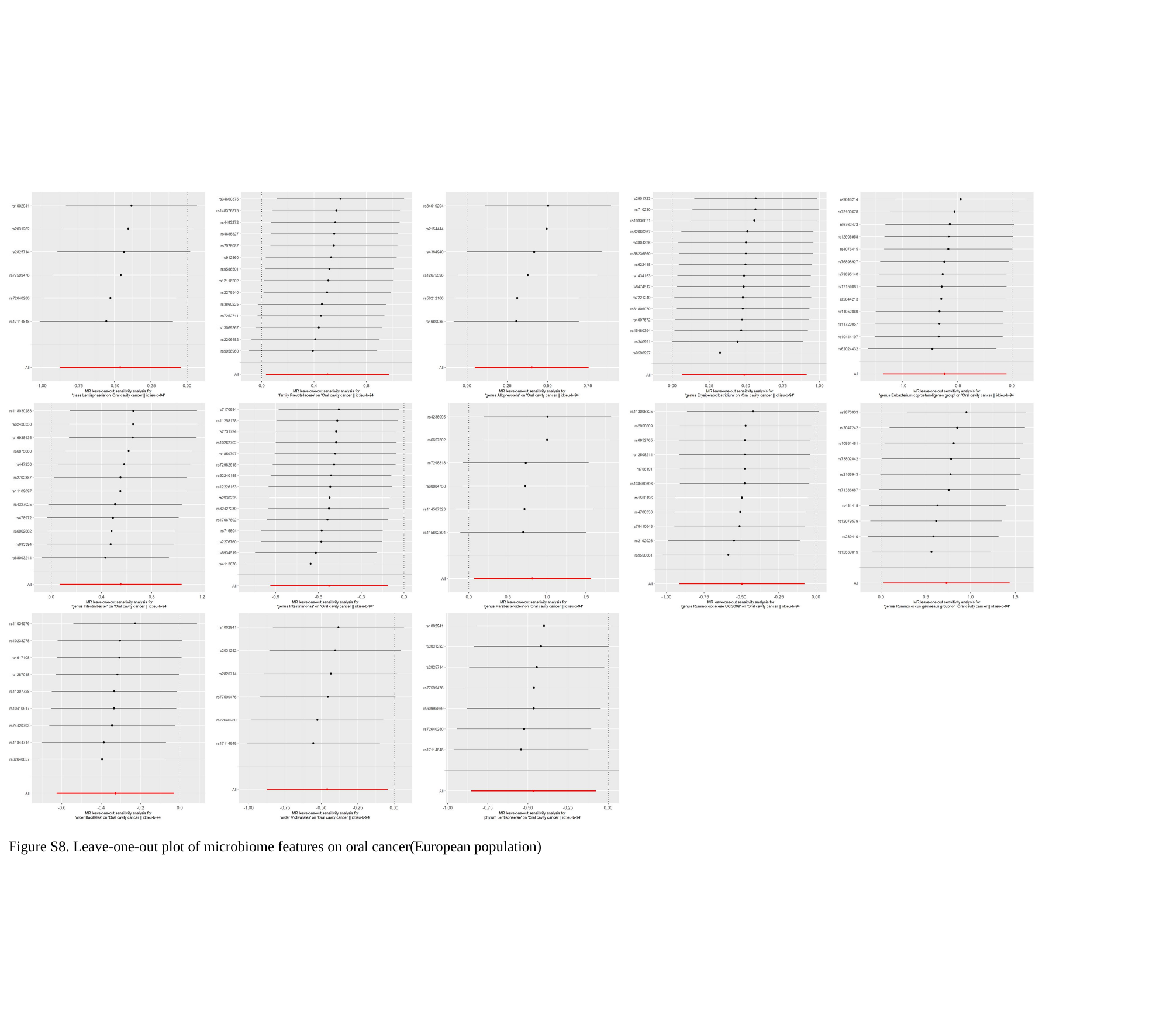

Figure S8. Leave-one-out plot of microbiome features on oral cancer(European population)

## Slide 9
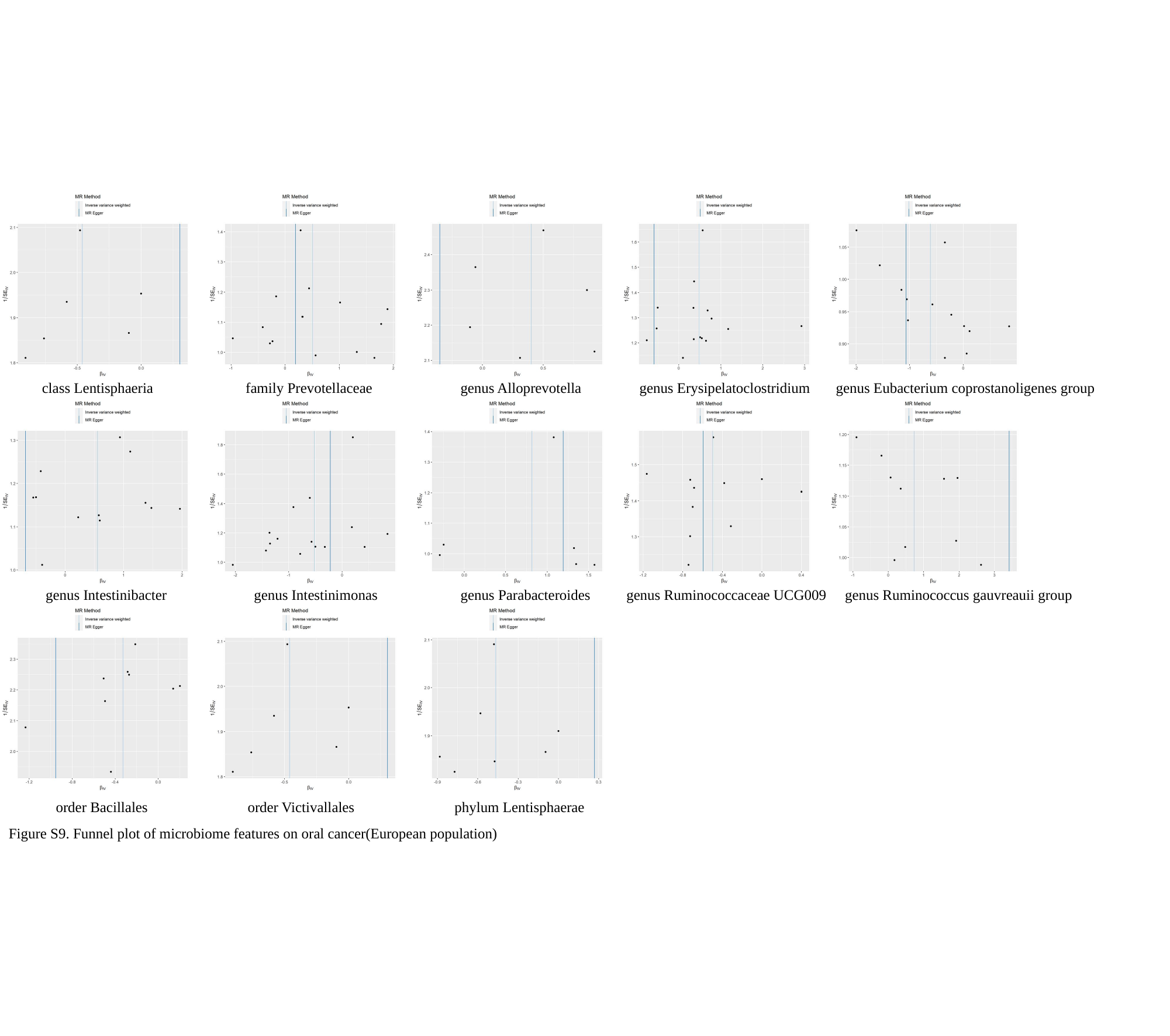

class Lentisphaeria
family Prevotellaceae
genus Alloprevotella
genus Erysipelatoclostridium
genus Eubacterium coprostanoligenes group
genus Intestinibacter
genus Intestinimonas
genus Parabacteroides
genus Ruminococcaceae UCG009
genus Ruminococcus gauvreauii group
order Bacillales
order Victivallales
phylum Lentisphaerae
Figure S9. Funnel plot of microbiome features on oral cancer(European population)

## Slide 10
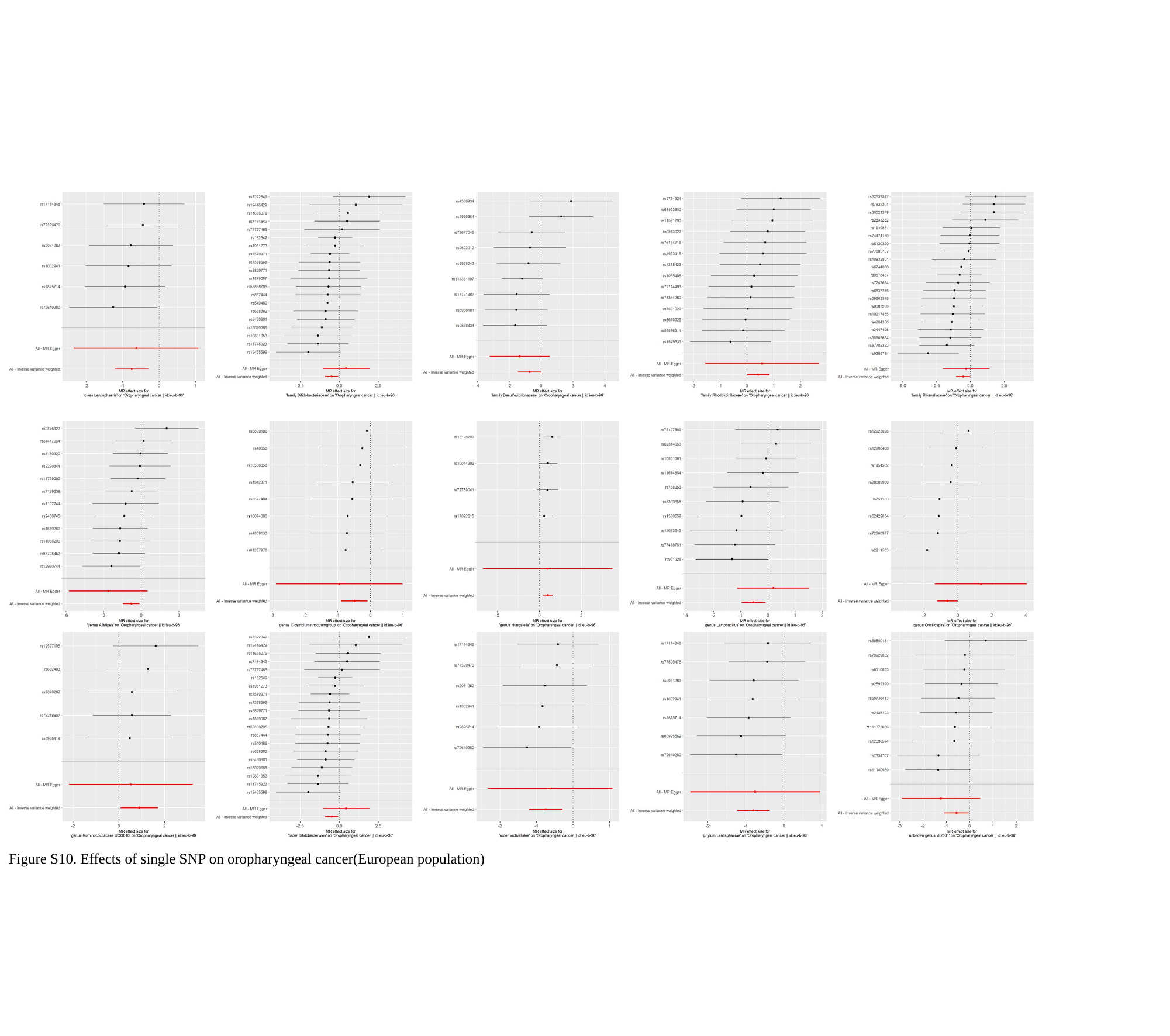

Figure S10. Effects of single SNP on oropharyngeal cancer(European population)

## Slide 11
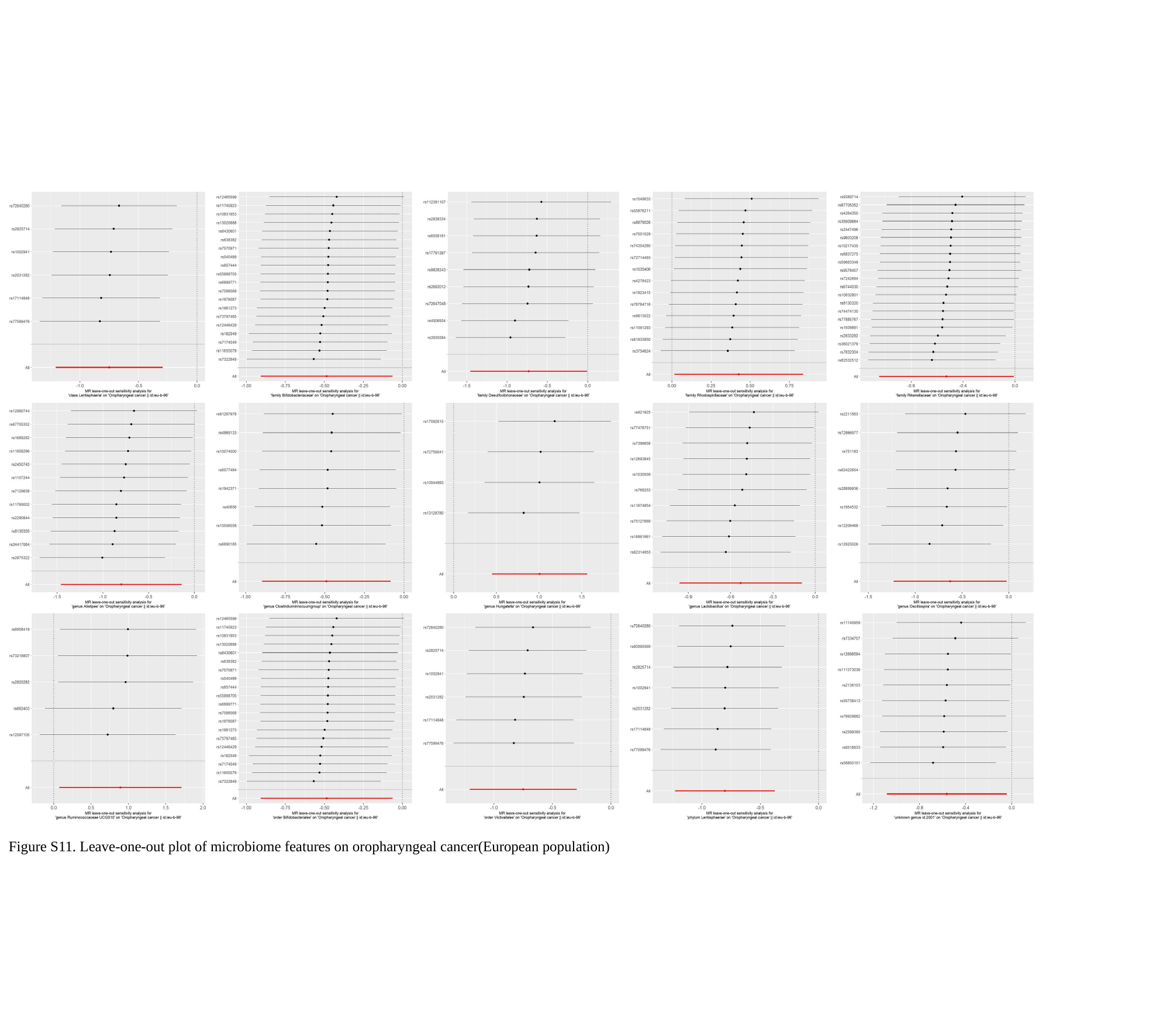

Figure S11. Leave-one-out plot of microbiome features on oropharyngeal cancer(European population)

## Slide 12
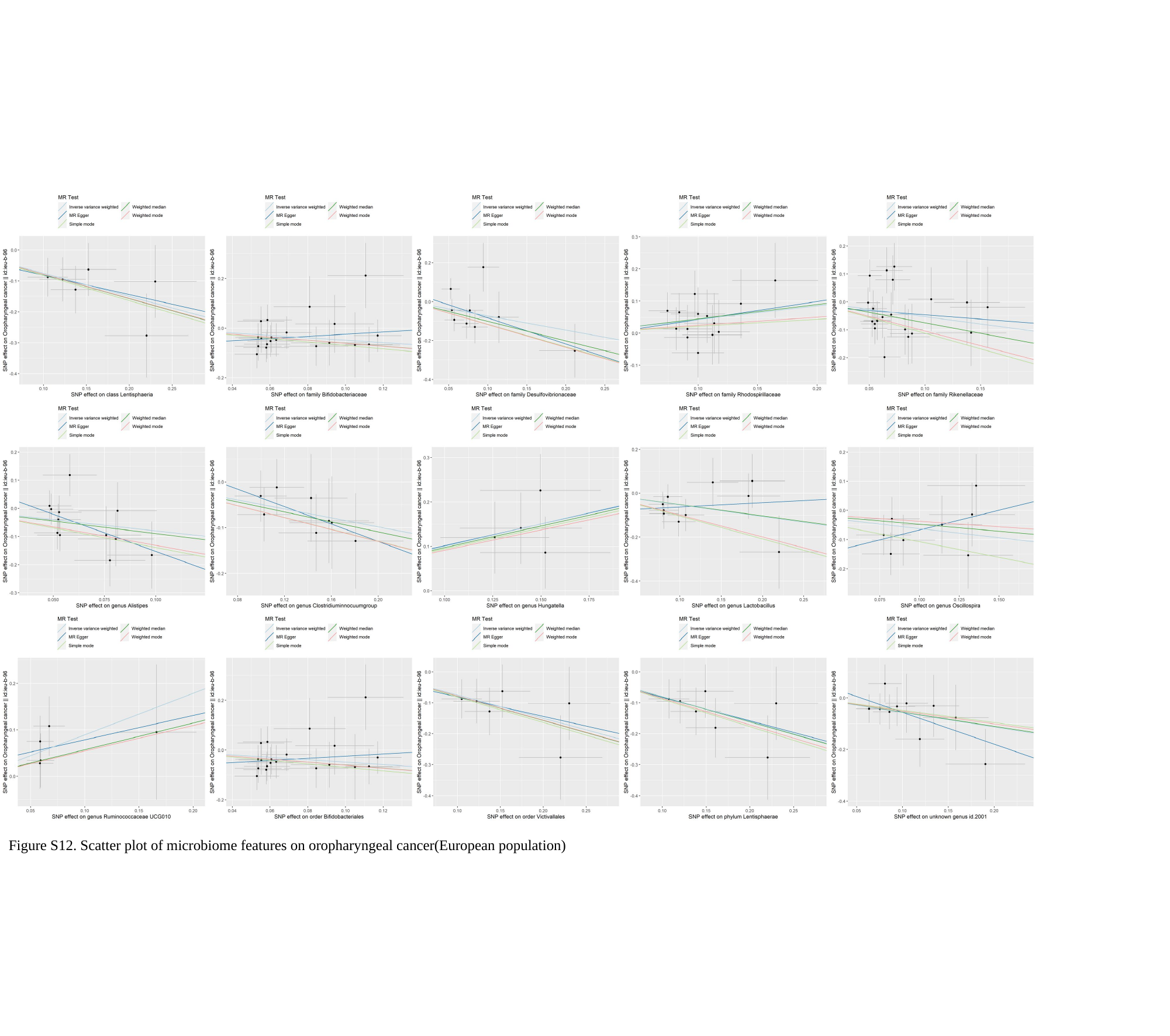

Figure S12. Scatter plot of microbiome features on oropharyngeal cancer(European population)

## Slide 13
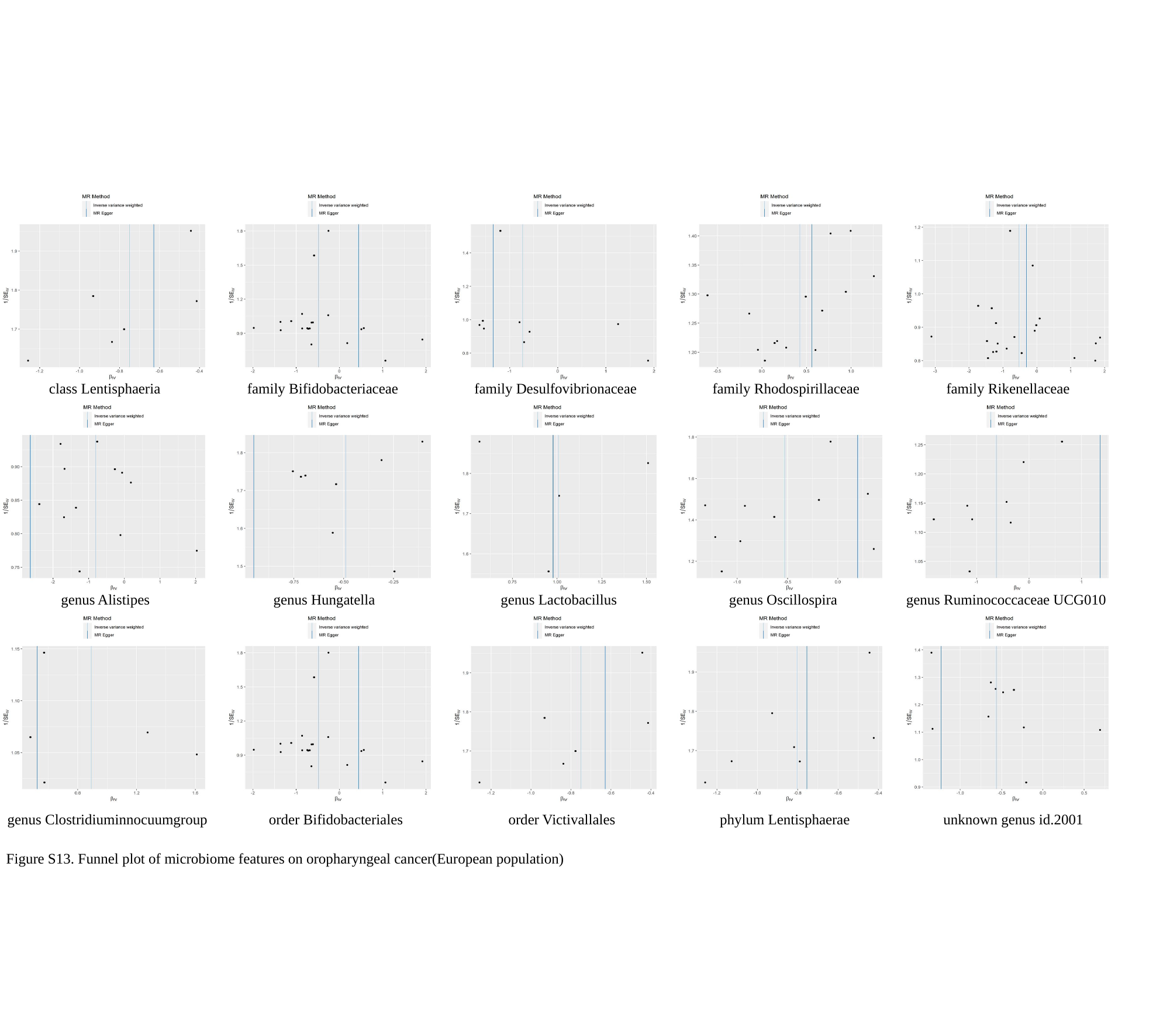

class Lentisphaeria
family Bifidobacteriaceae
family Desulfovibrionaceae
family Rhodospirillaceae
family Rikenellaceae
genus Alistipes
genus Hungatella
genus Lactobacillus
genus Oscillospira
genus Ruminococcaceae UCG010
genus Clostridiuminnocuumgroup
order Bifidobacteriales
order Victivallales
phylum Lentisphaerae
unknown genus id.2001
Figure S13. Funnel plot of microbiome features on oropharyngeal cancer(European population)

## Slide 14
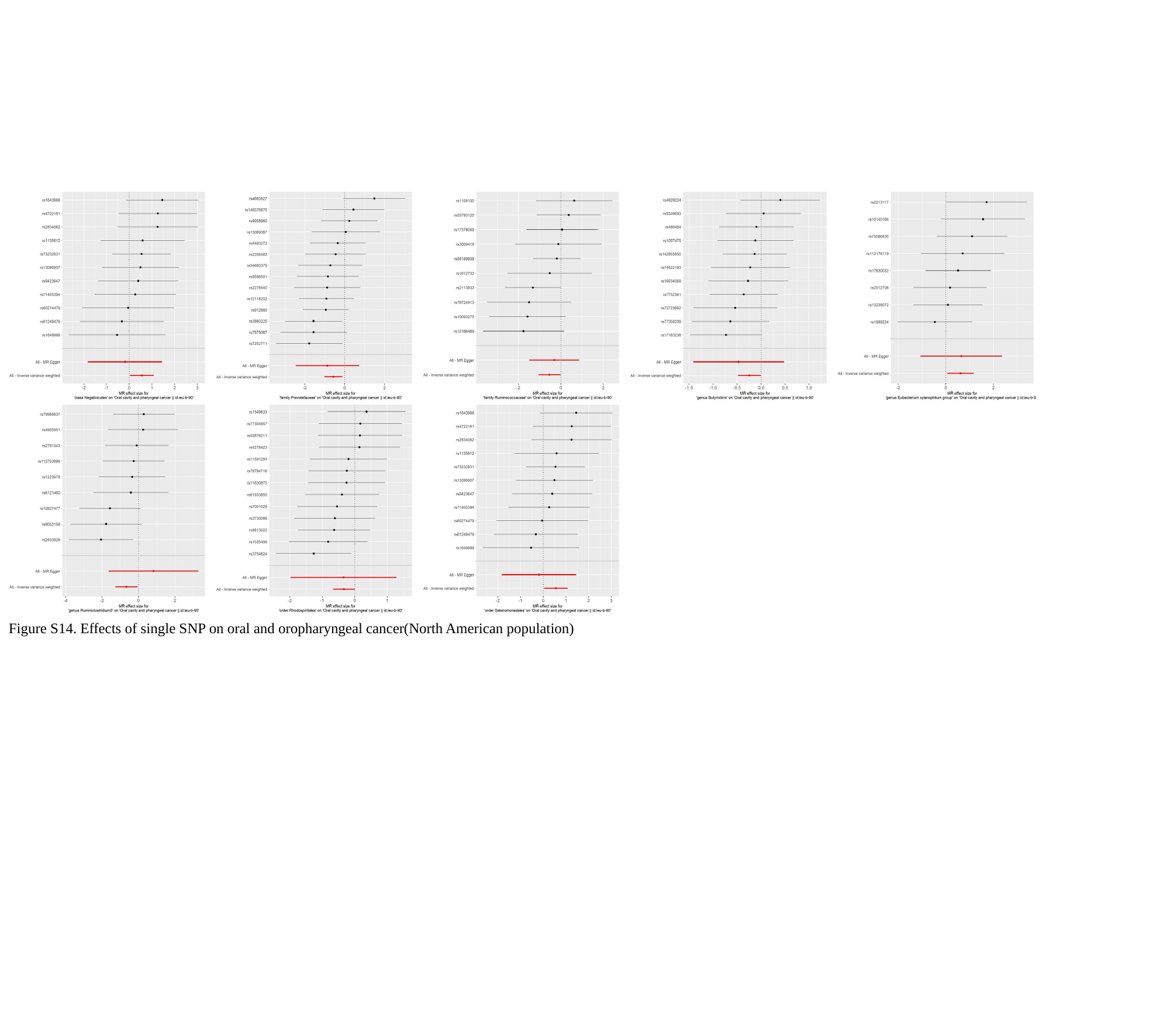

Figure S14. Effects of single SNP on oral and oropharyngeal cancer(North American population)

## Slide 15
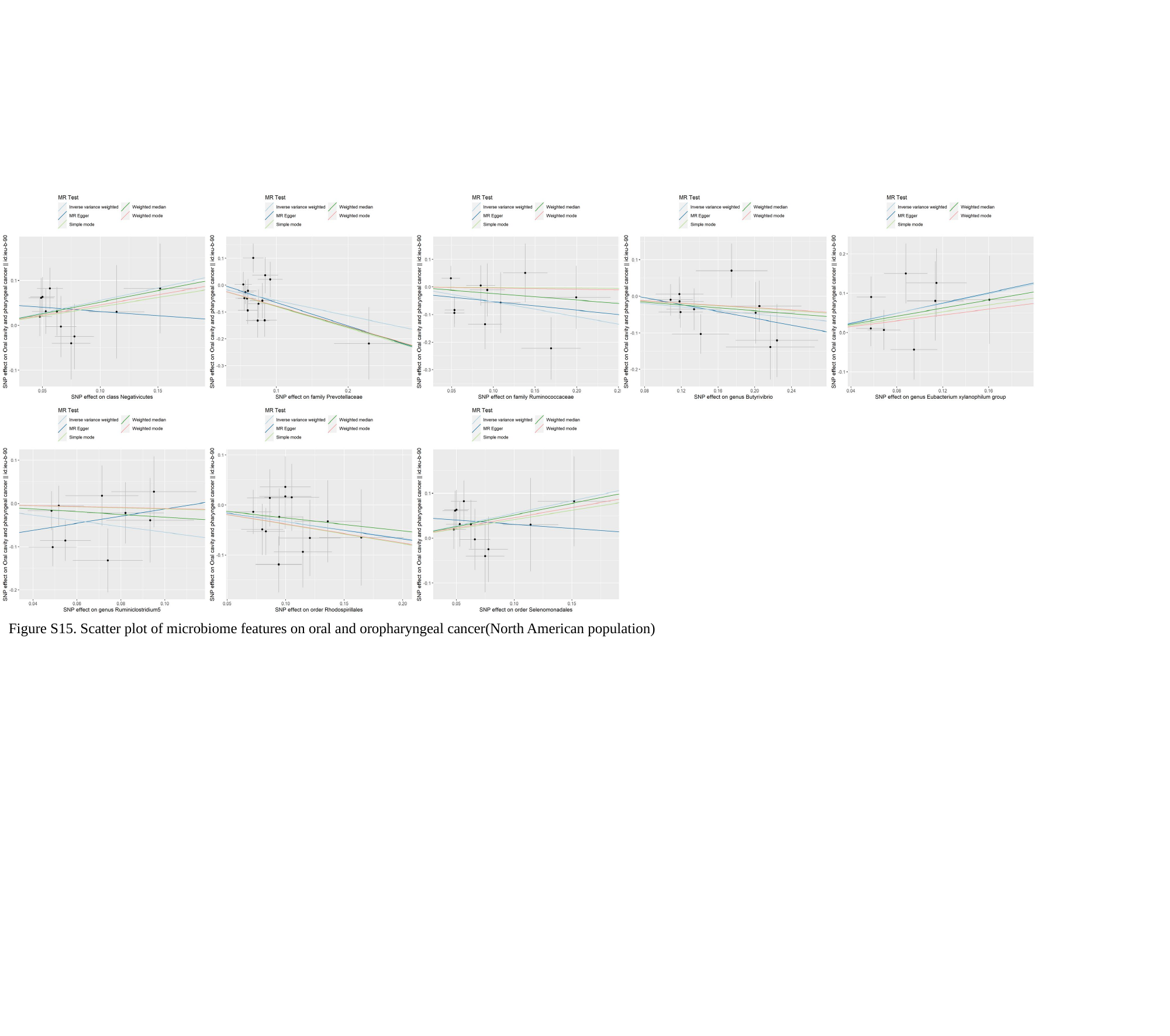

Figure S15. Scatter plot of microbiome features on oral and oropharyngeal cancer(North American population)

## Slide 16
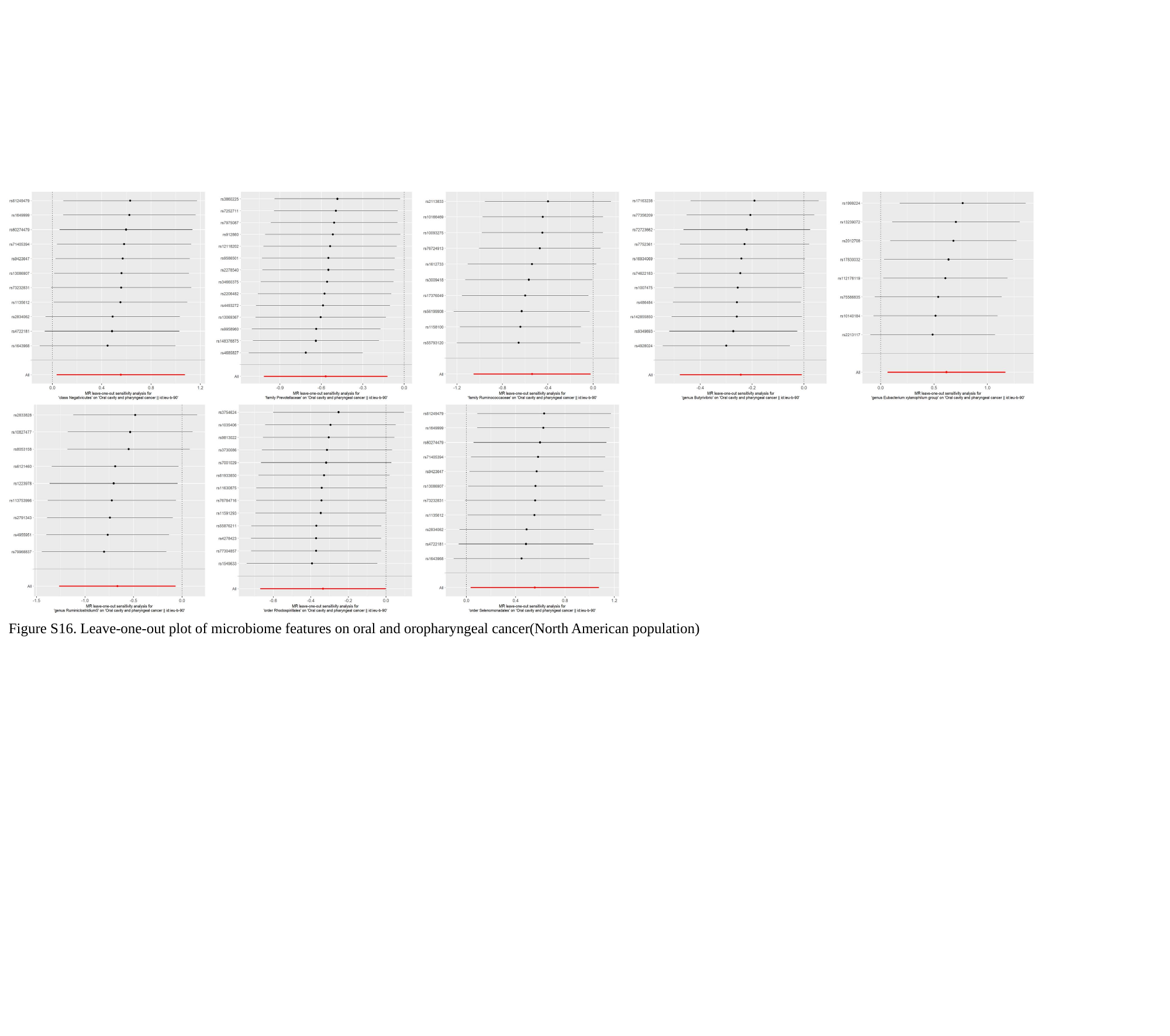

Figure S16. Leave-one-out plot of microbiome features on oral and oropharyngeal cancer(North American population)

## Slide 17
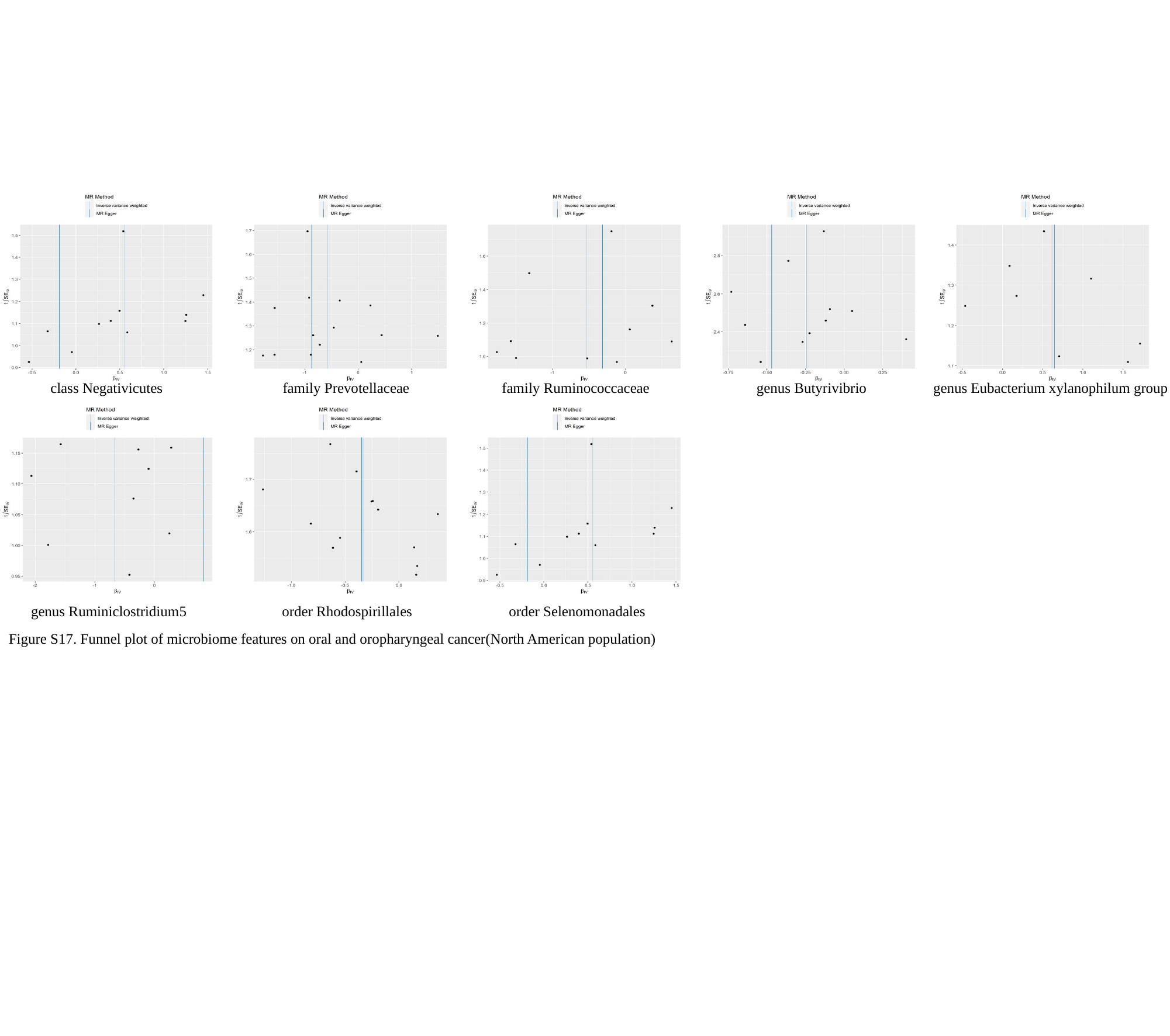

class Negativicutes
family Prevotellaceae
family Ruminococcaceae
genus Butyrivibrio
genus Eubacterium xylanophilum group
genus Ruminiclostridium5
order Rhodospirillales
order Selenomonadales
Figure S17. Funnel plot of microbiome features on oral and oropharyngeal cancer(North American population)

## Slide 18
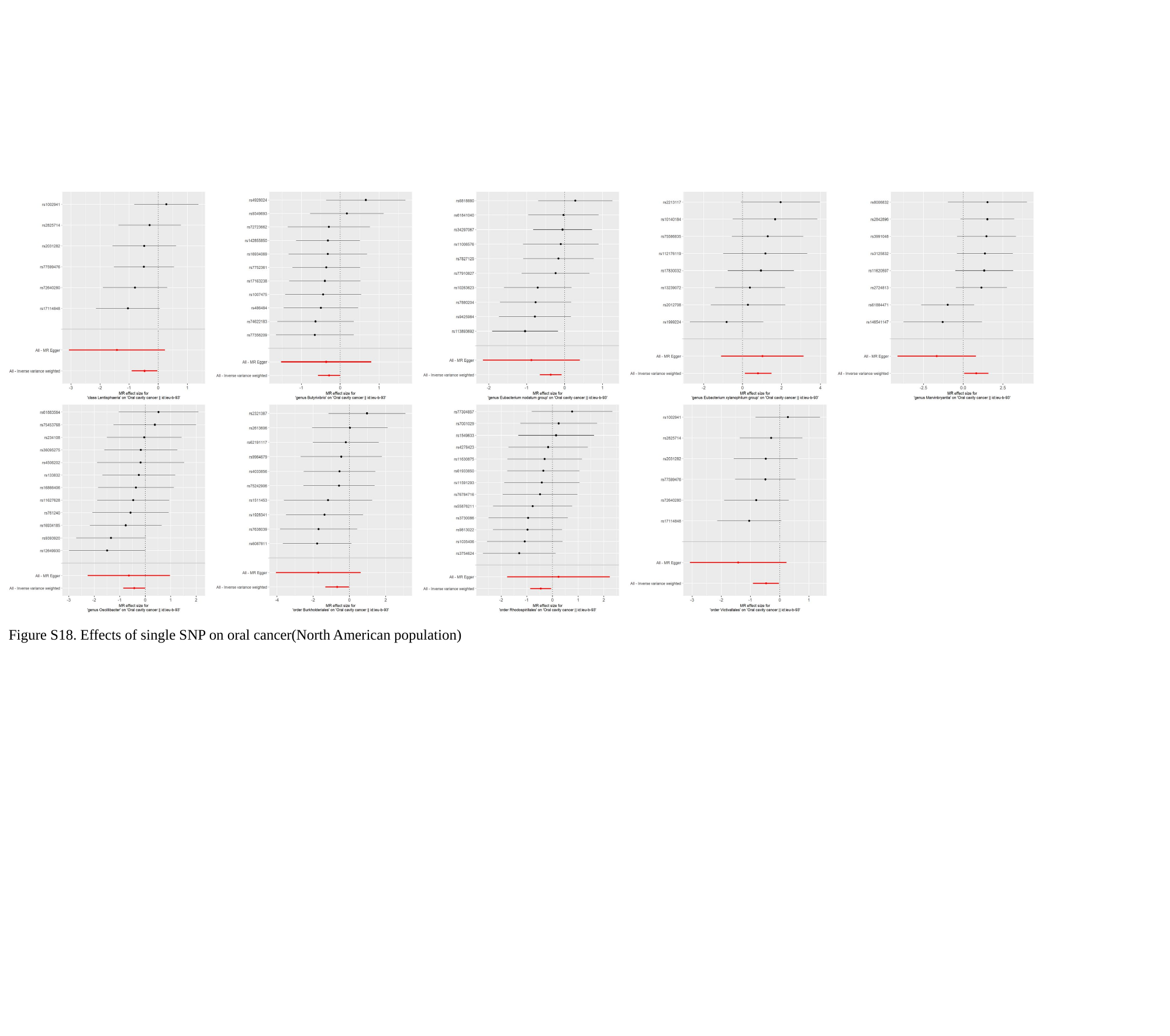

Figure S18. Effects of single SNP on oral cancer(North American population)

## Slide 19
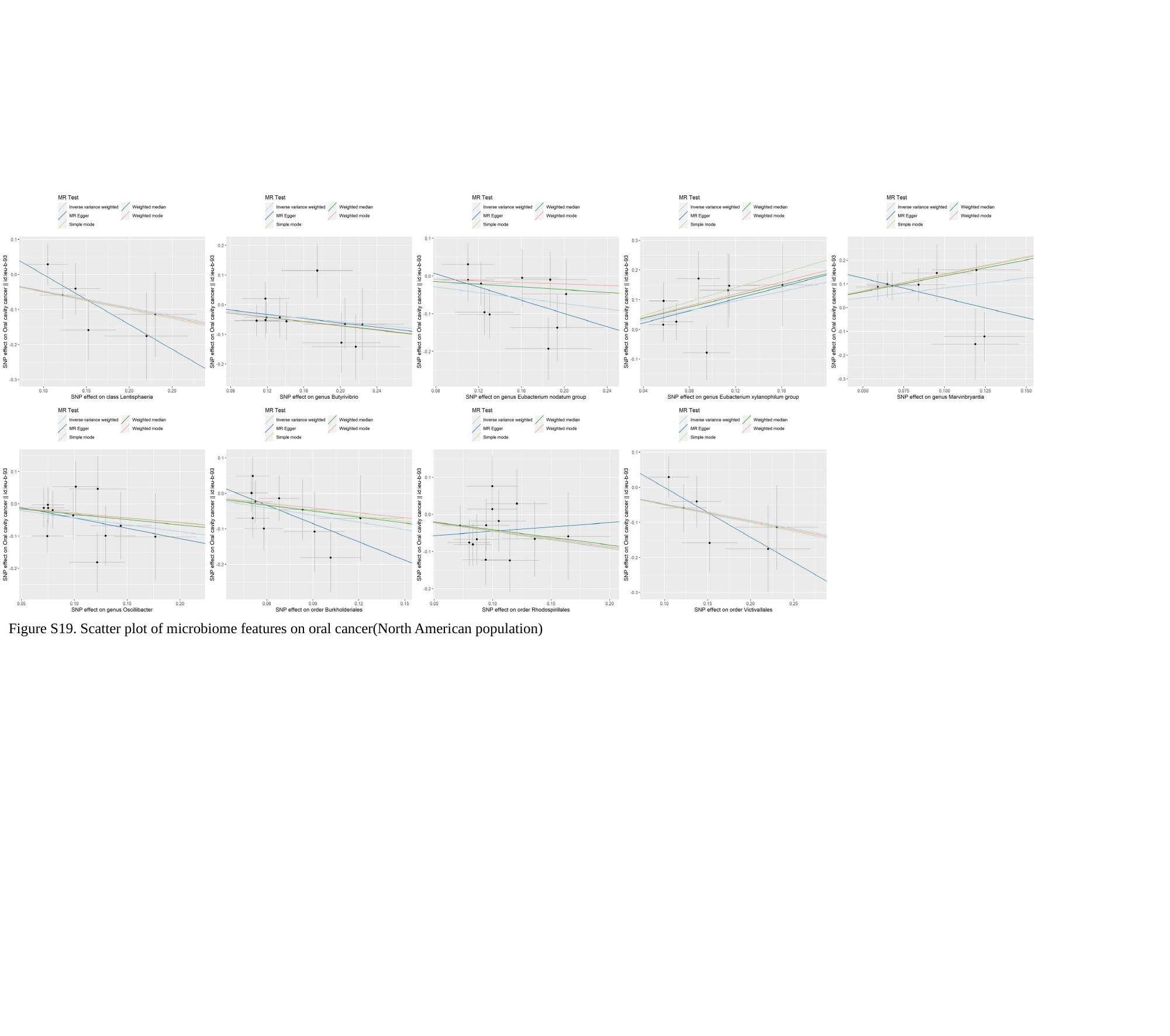

Figure S19. Scatter plot of microbiome features on oral cancer(North American population)

## Slide 20
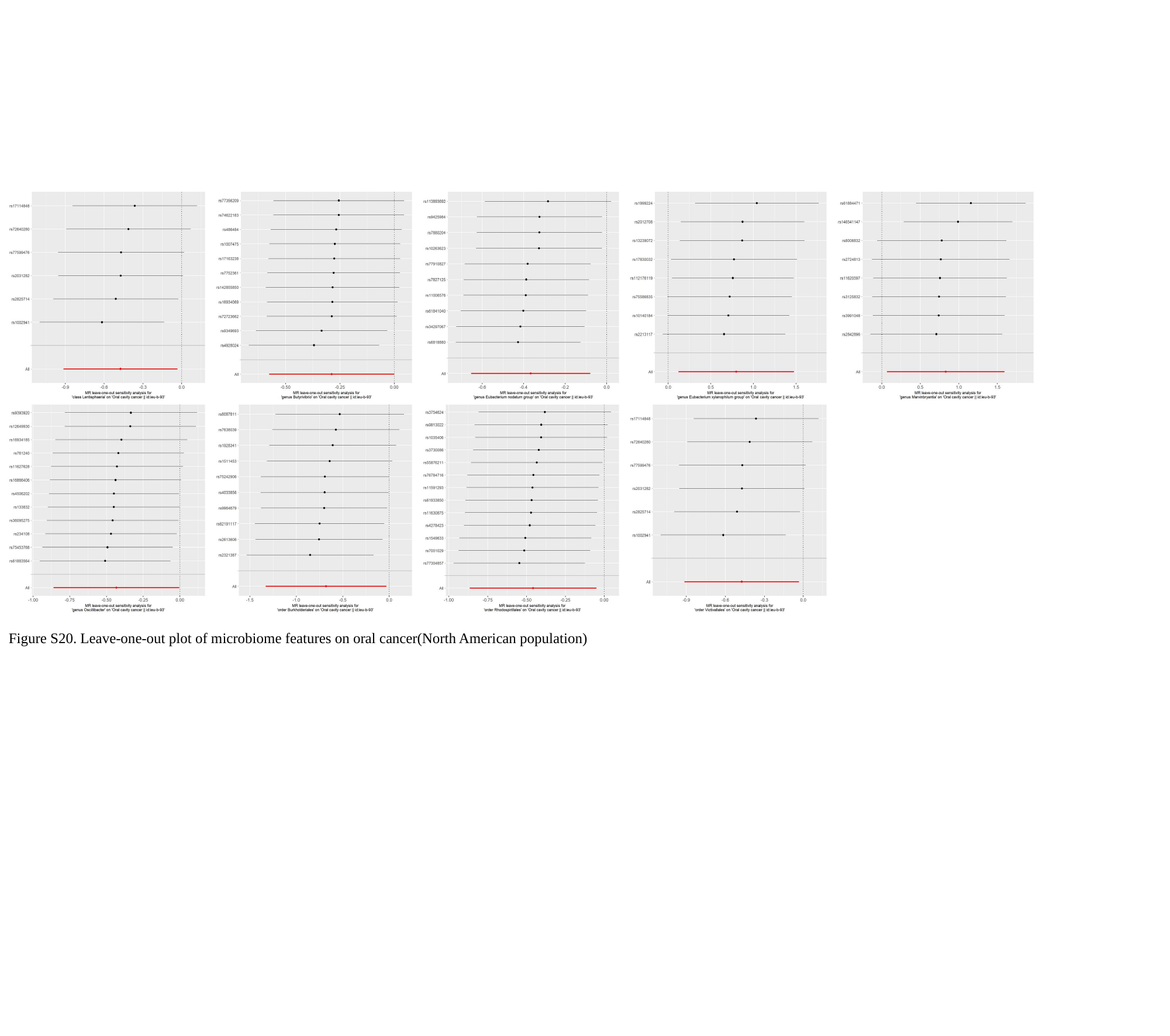

Figure S20. Leave-one-out plot of microbiome features on oral cancer(North American population)

## Slide 21
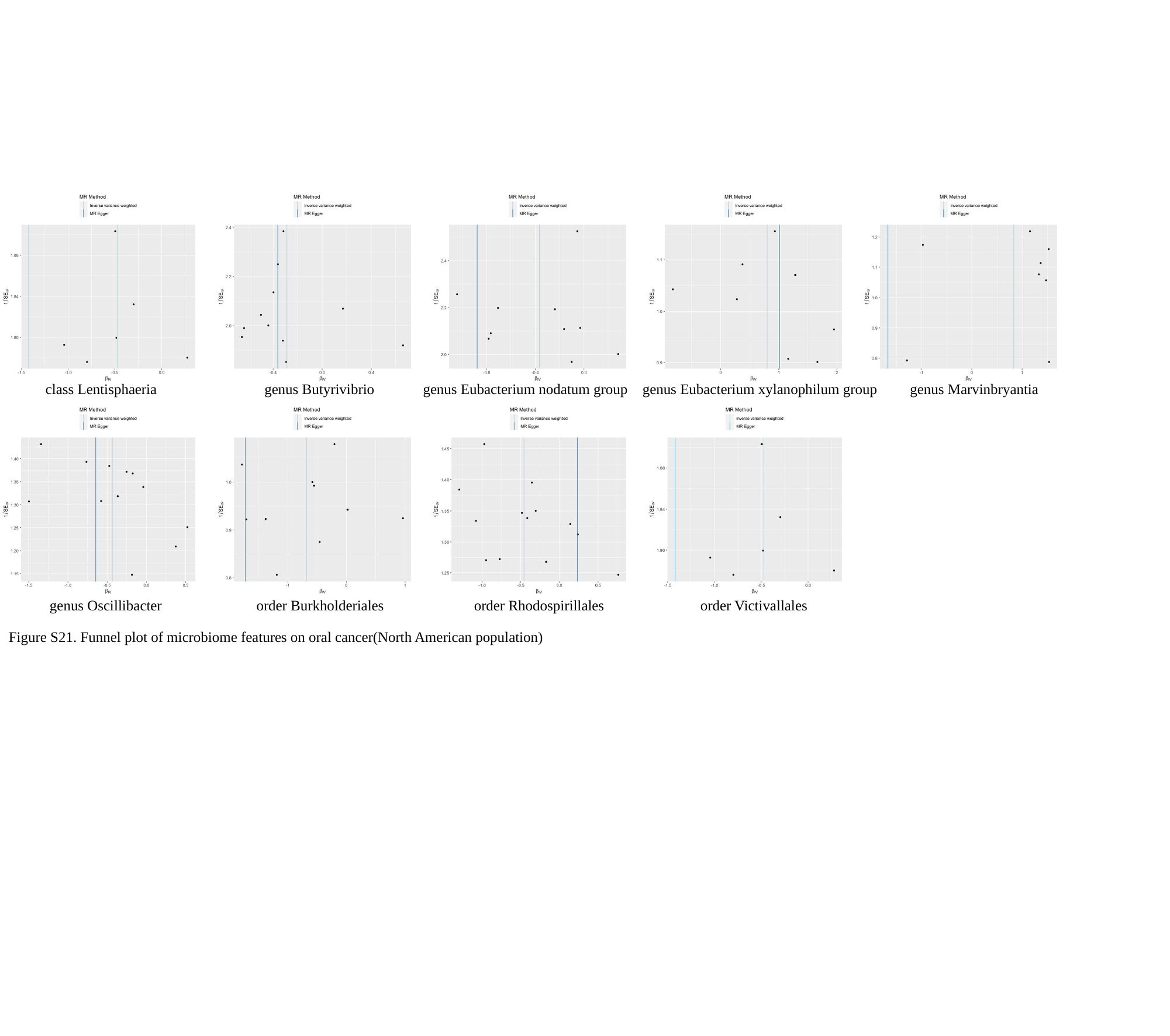

class Lentisphaeria
genus Butyrivibrio
genus Eubacterium nodatum group
genus Eubacterium xylanophilum group
genus Marvinbryantia
genus Oscillibacter
order Burkholderiales
order Rhodospirillales
order Victivallales
Figure S21. Funnel plot of microbiome features on oral cancer(North American population)

## Slide 22
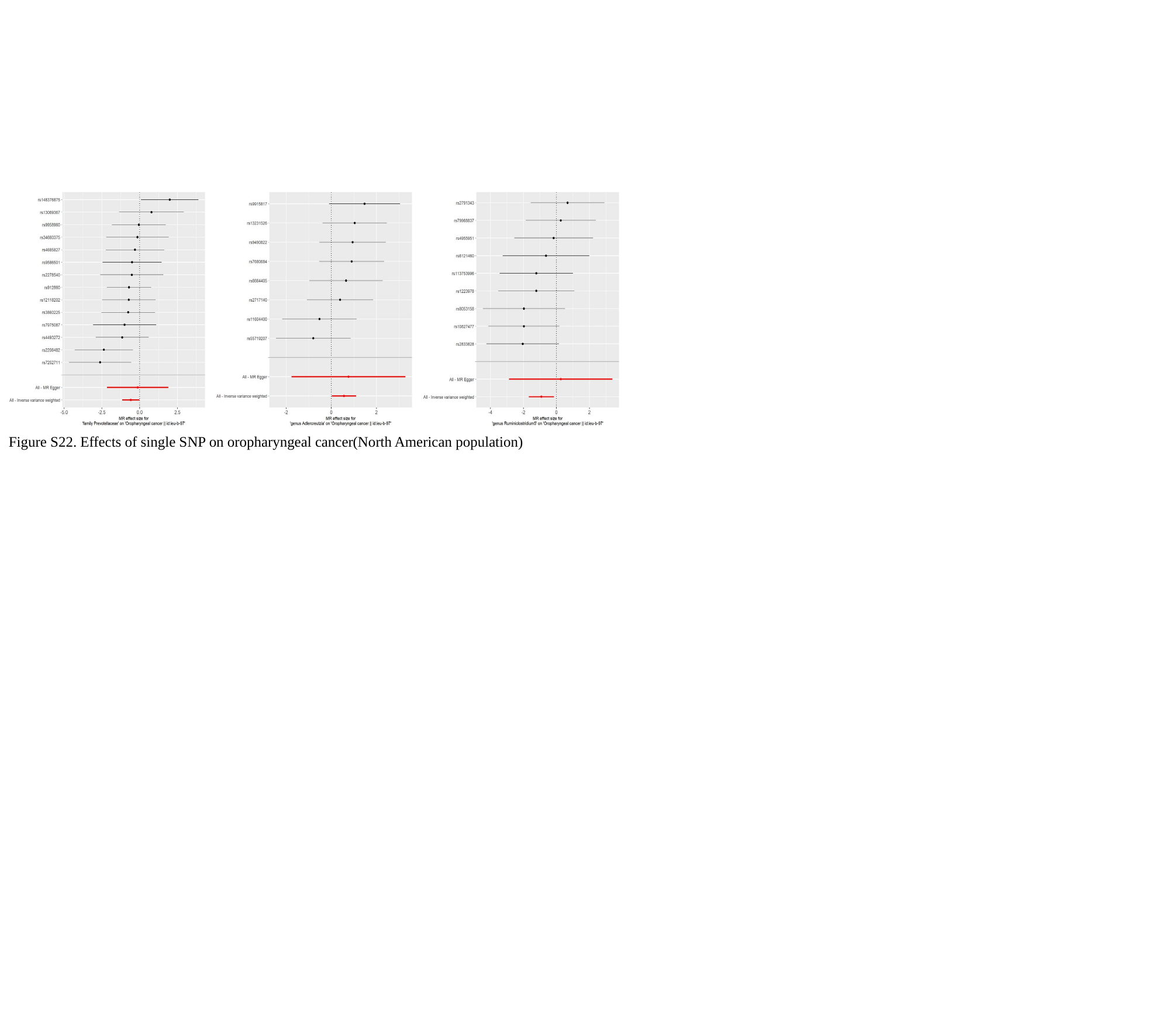

Figure S22. Effects of single SNP on oropharyngeal cancer(North American population)

## Slide 23
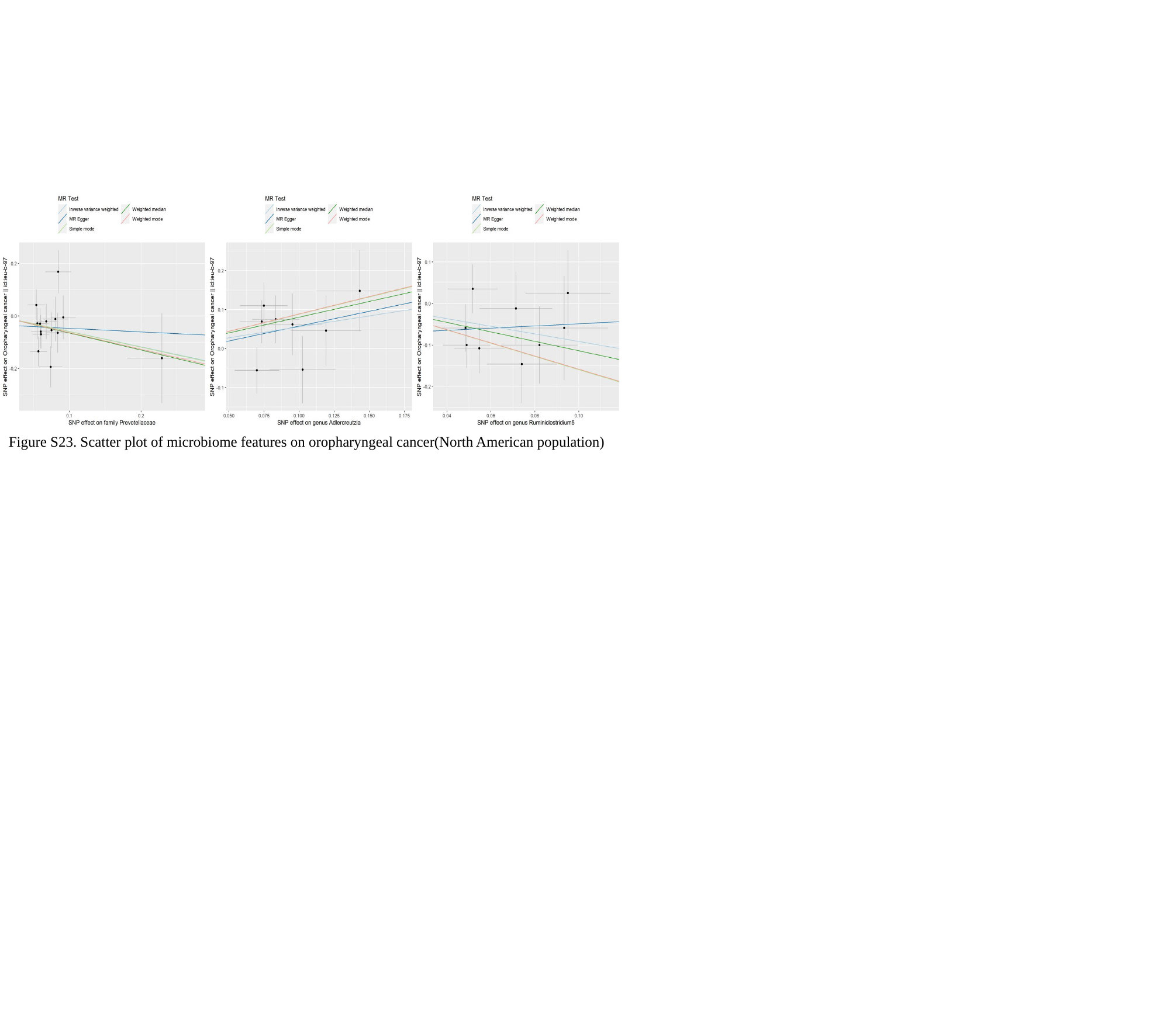

Figure S23. Scatter plot of microbiome features on oropharyngeal cancer(North American population)

## Slide 24
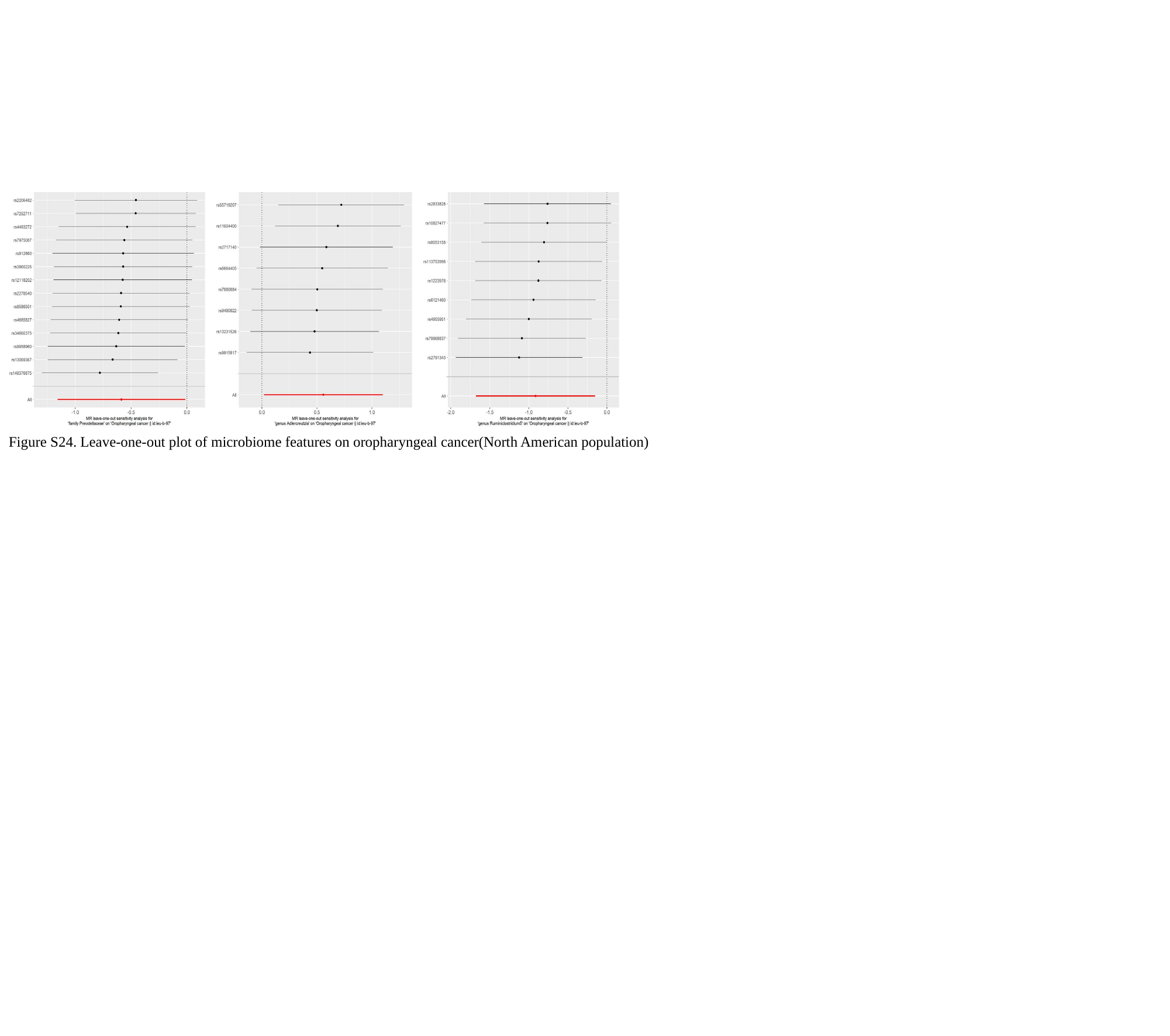

Figure S24. Leave-one-out plot of microbiome features on oropharyngeal cancer(North American population)

## Slide 25
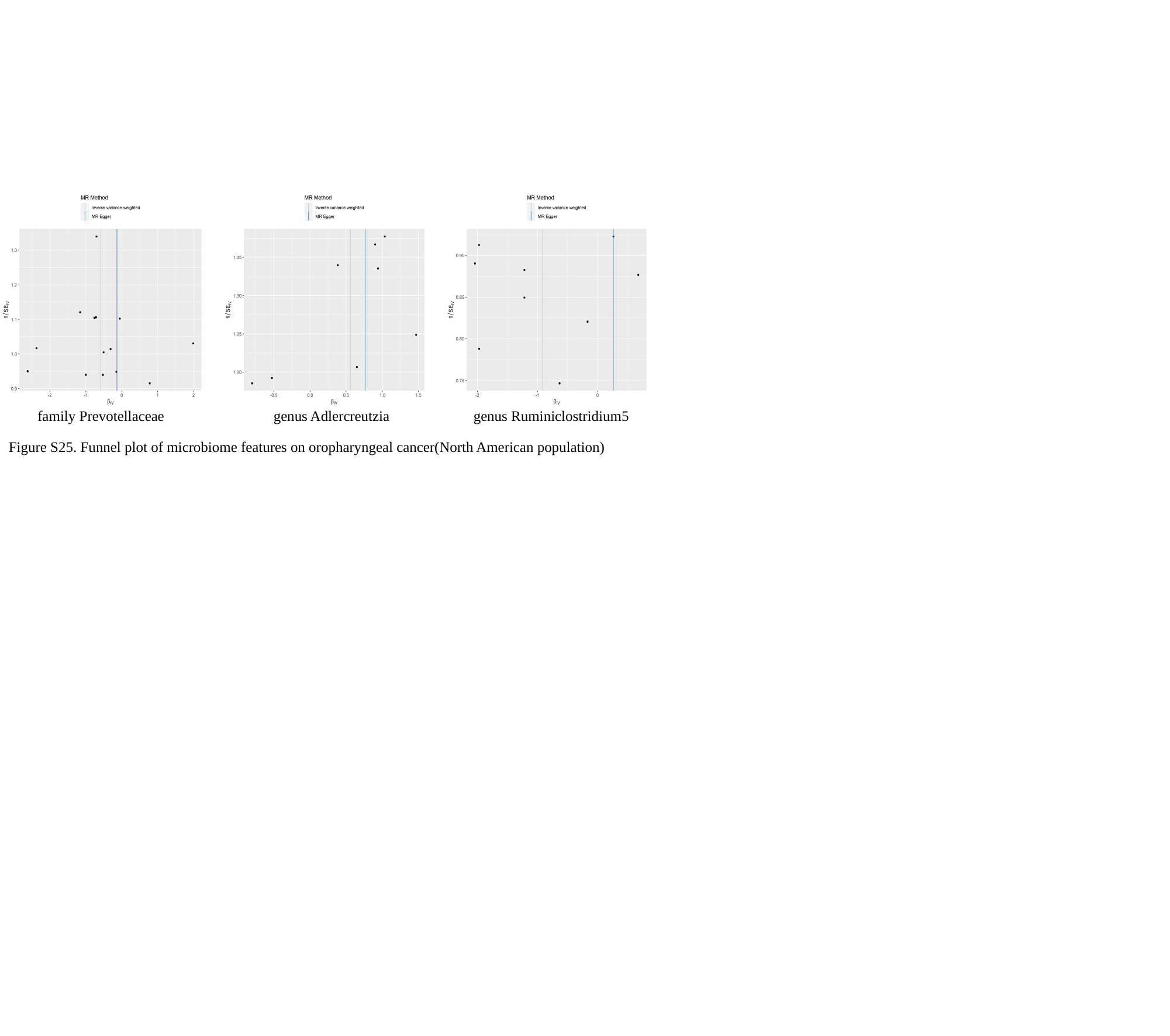

family Prevotellaceae
genus Adlercreutzia
genus Ruminiclostridium5
Figure S25. Funnel plot of microbiome features on oropharyngeal cancer(North American population)

## Slide 26
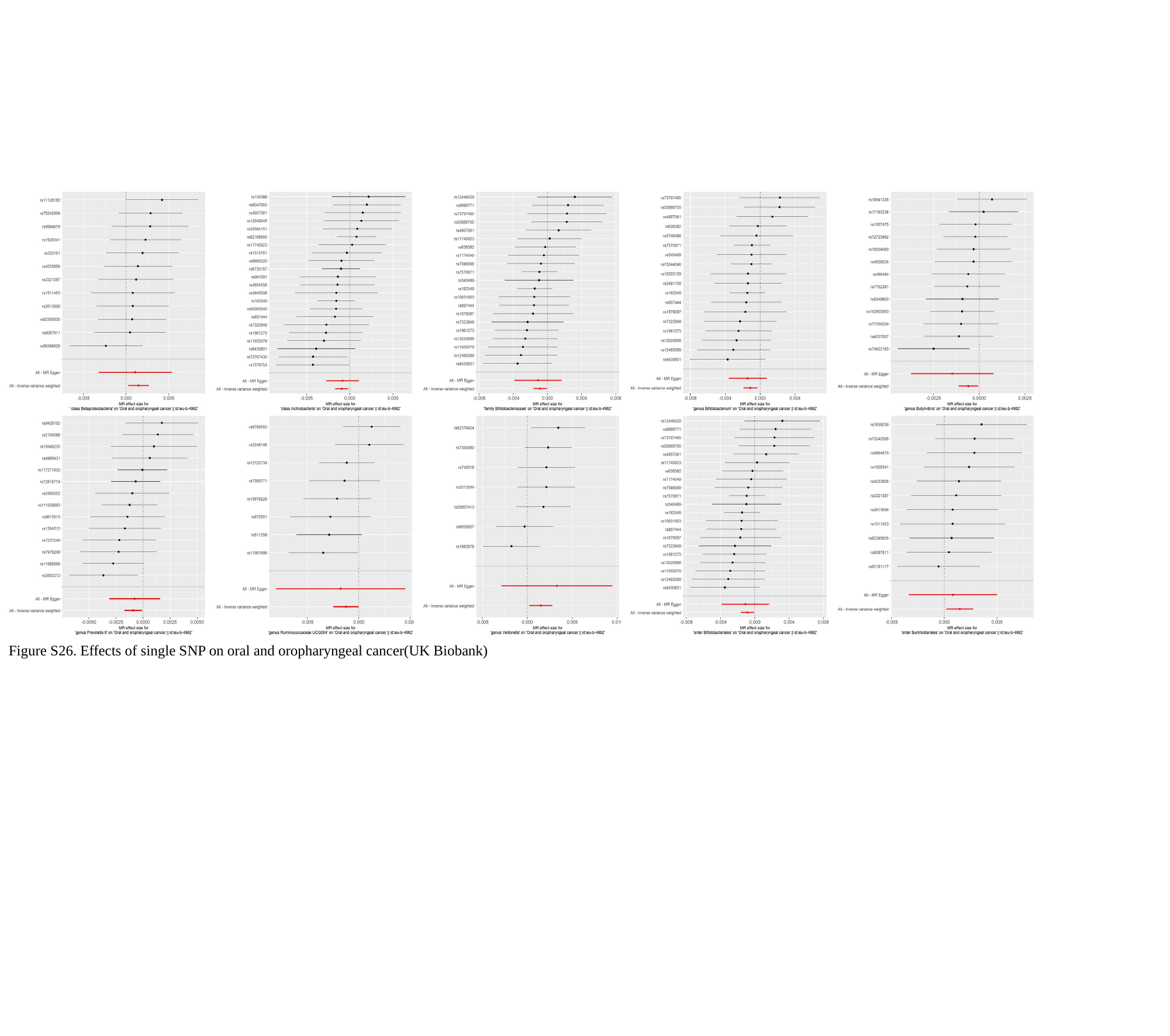

Figure S26. Effects of single SNP on oral and oropharyngeal cancer(UK Biobank)

## Slide 27
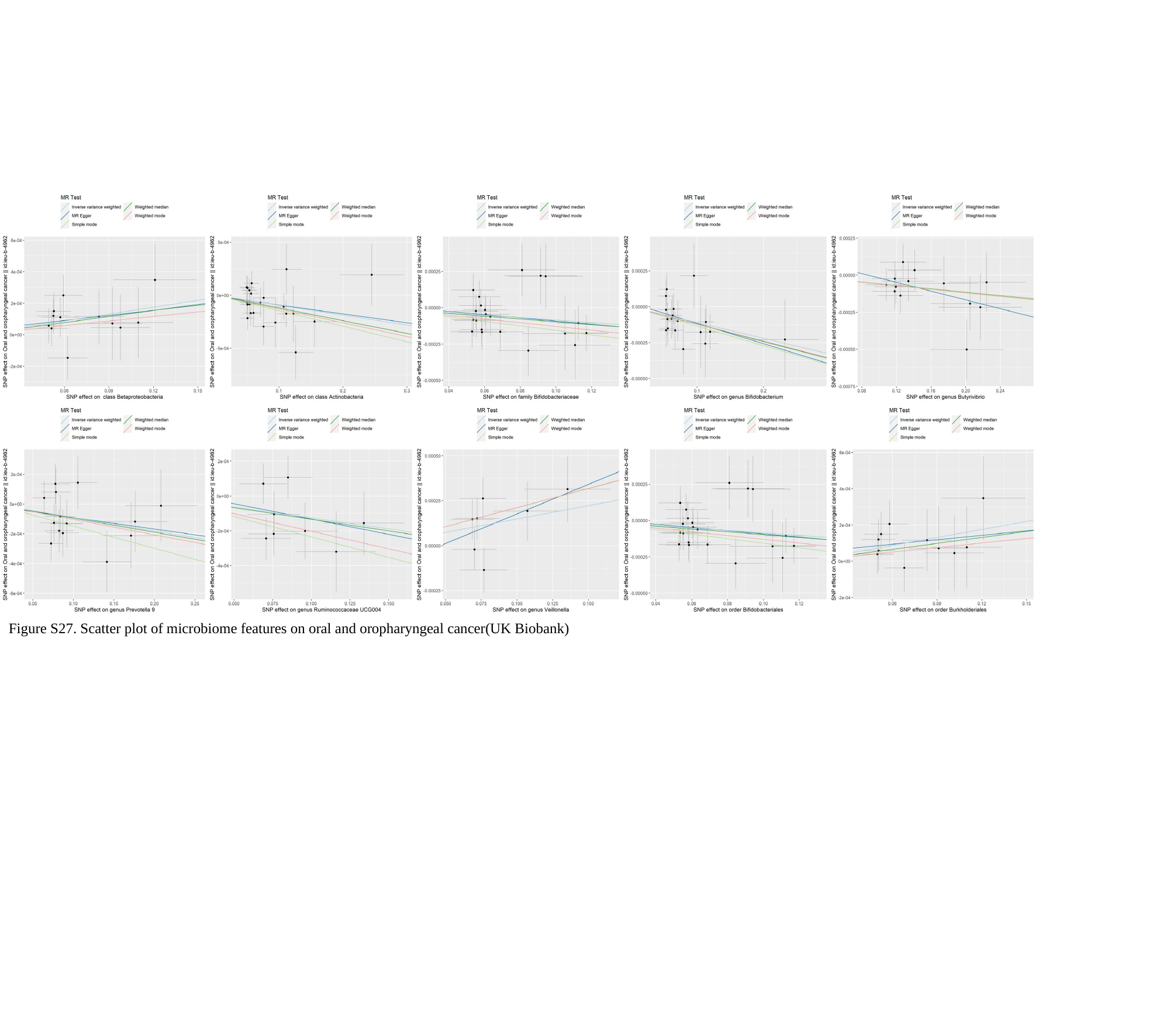

Figure S27. Scatter plot of microbiome features on oral and oropharyngeal cancer(UK Biobank)

## Slide 28
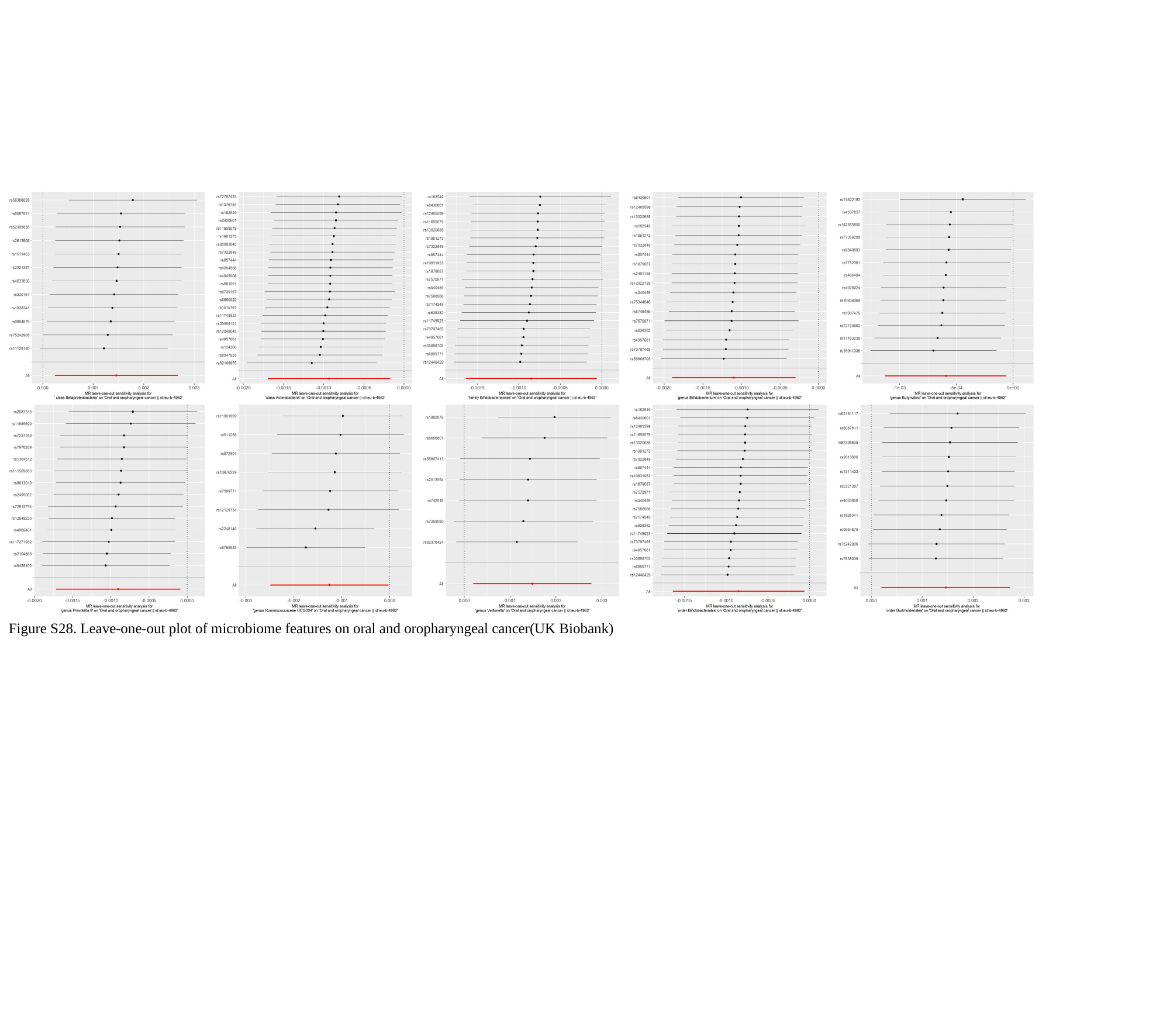

Figure S28. Leave-one-out plot of microbiome features on oral and oropharyngeal cancer(UK Biobank)

## Slide 29
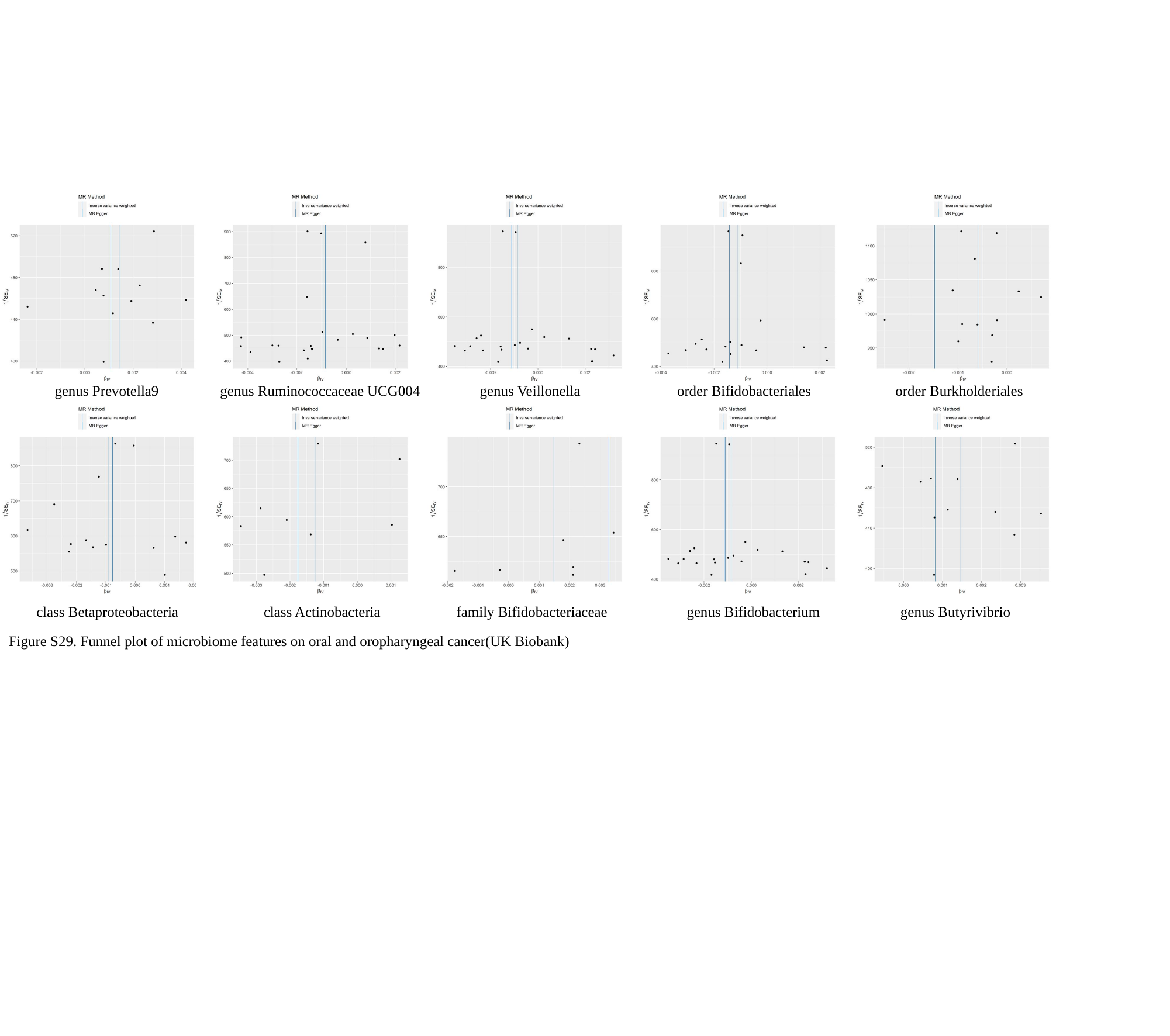

genus Prevotella9
genus Ruminococcaceae UCG004
genus Veillonella
order Bifidobacteriales
order Burkholderiales
class Betaproteobacteria
class Actinobacteria
family Bifidobacteriaceae
genus Bifidobacterium
genus Butyrivibrio
Figure S29. Funnel plot of microbiome features on oral and oropharyngeal cancer(UK Biobank)

## Slide 30
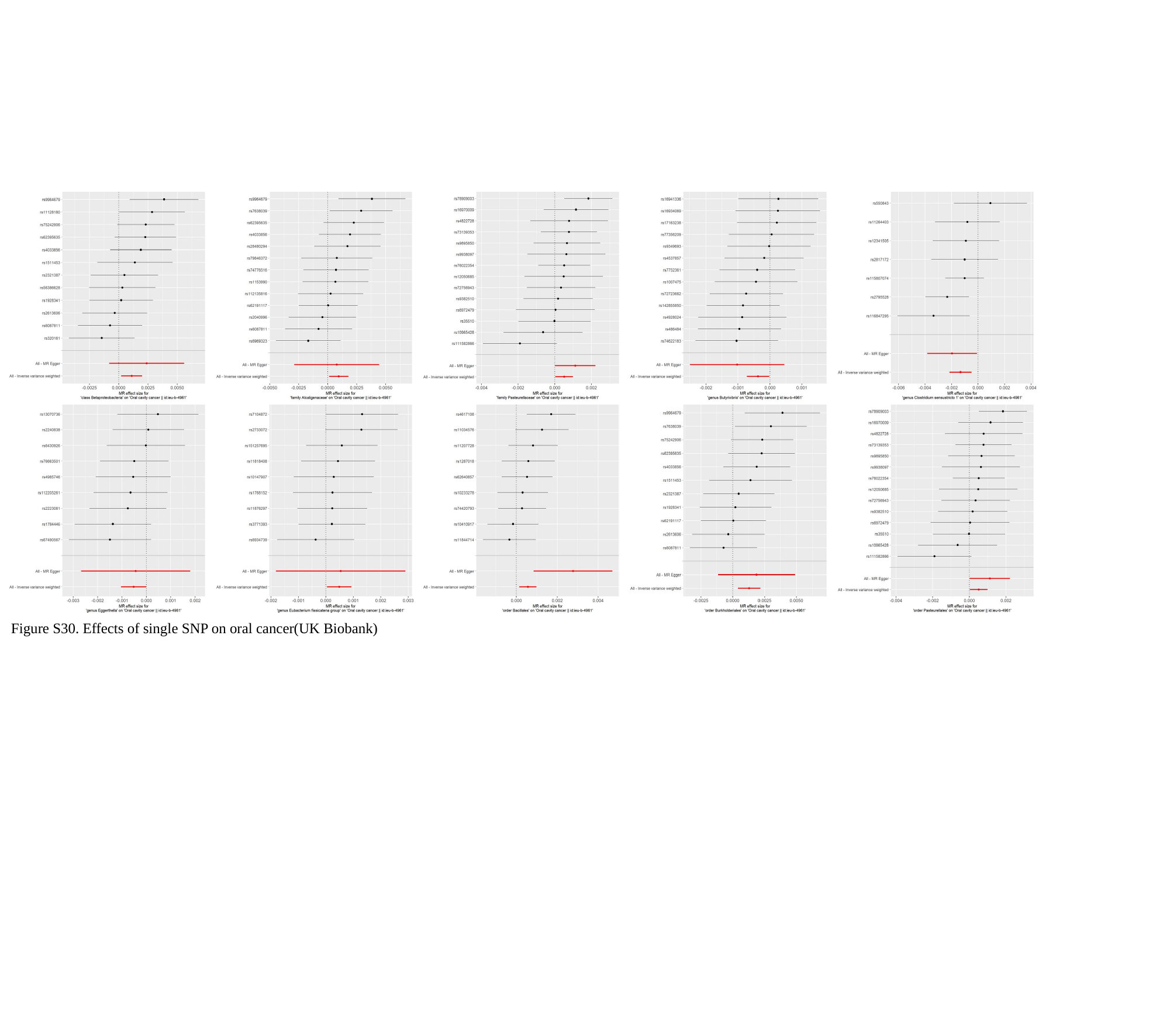

Figure S30. Effects of single SNP on oral cancer(UK Biobank)

## Slide 31
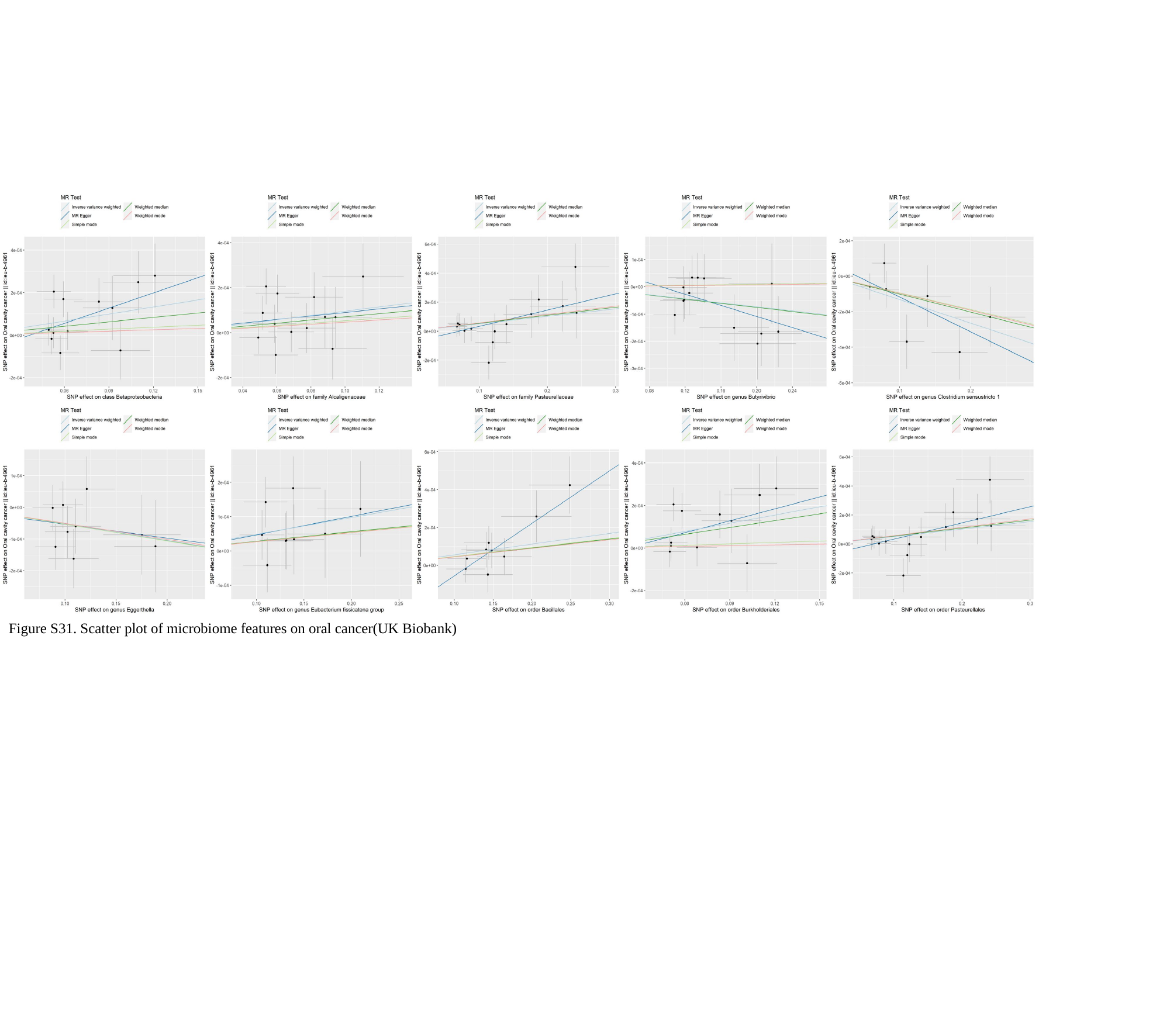

Figure S31. Scatter plot of microbiome features on oral cancer(UK Biobank)

## Slide 32
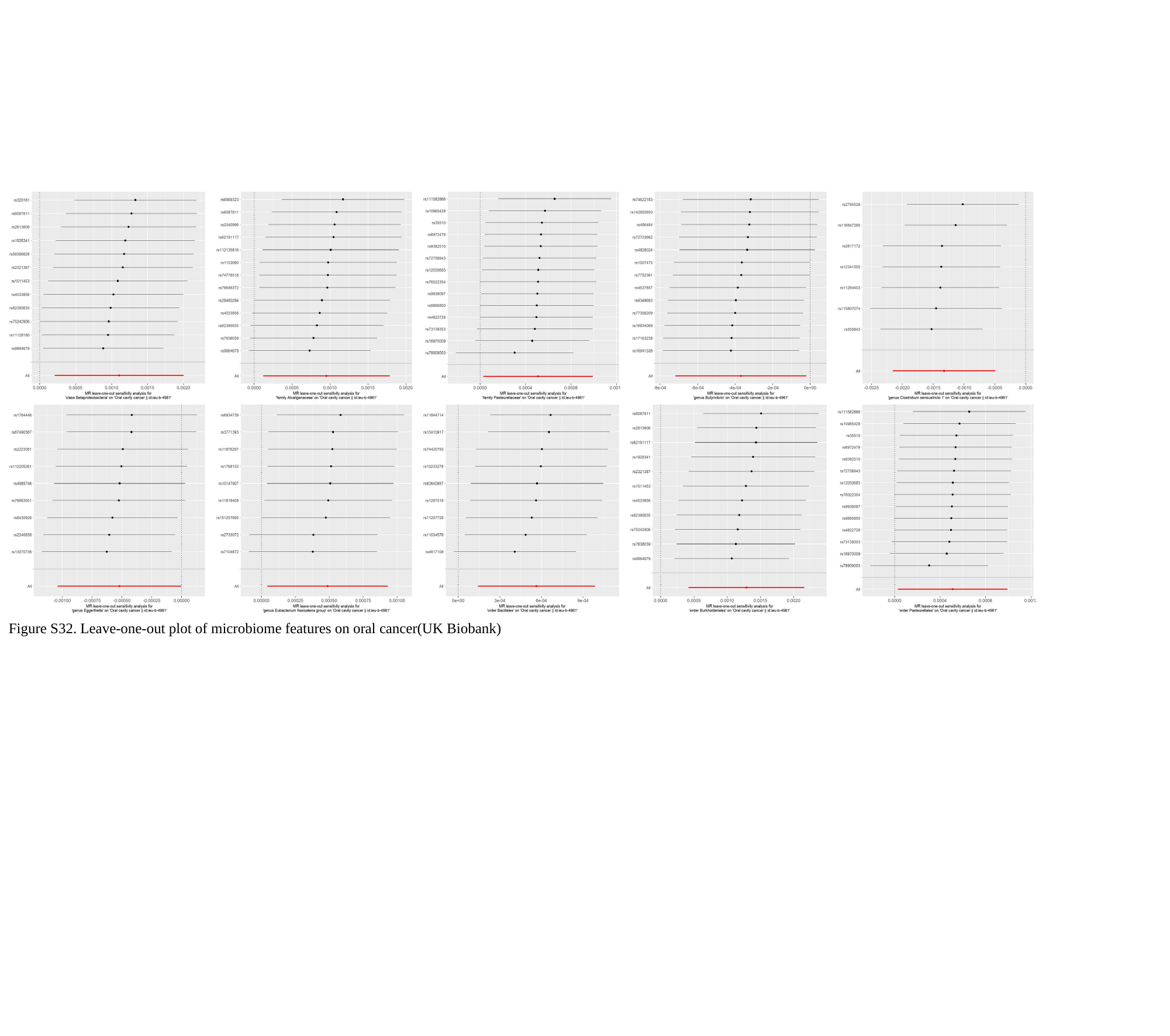

Figure S32. Leave-one-out plot of microbiome features on oral cancer(UK Biobank)

## Slide 33
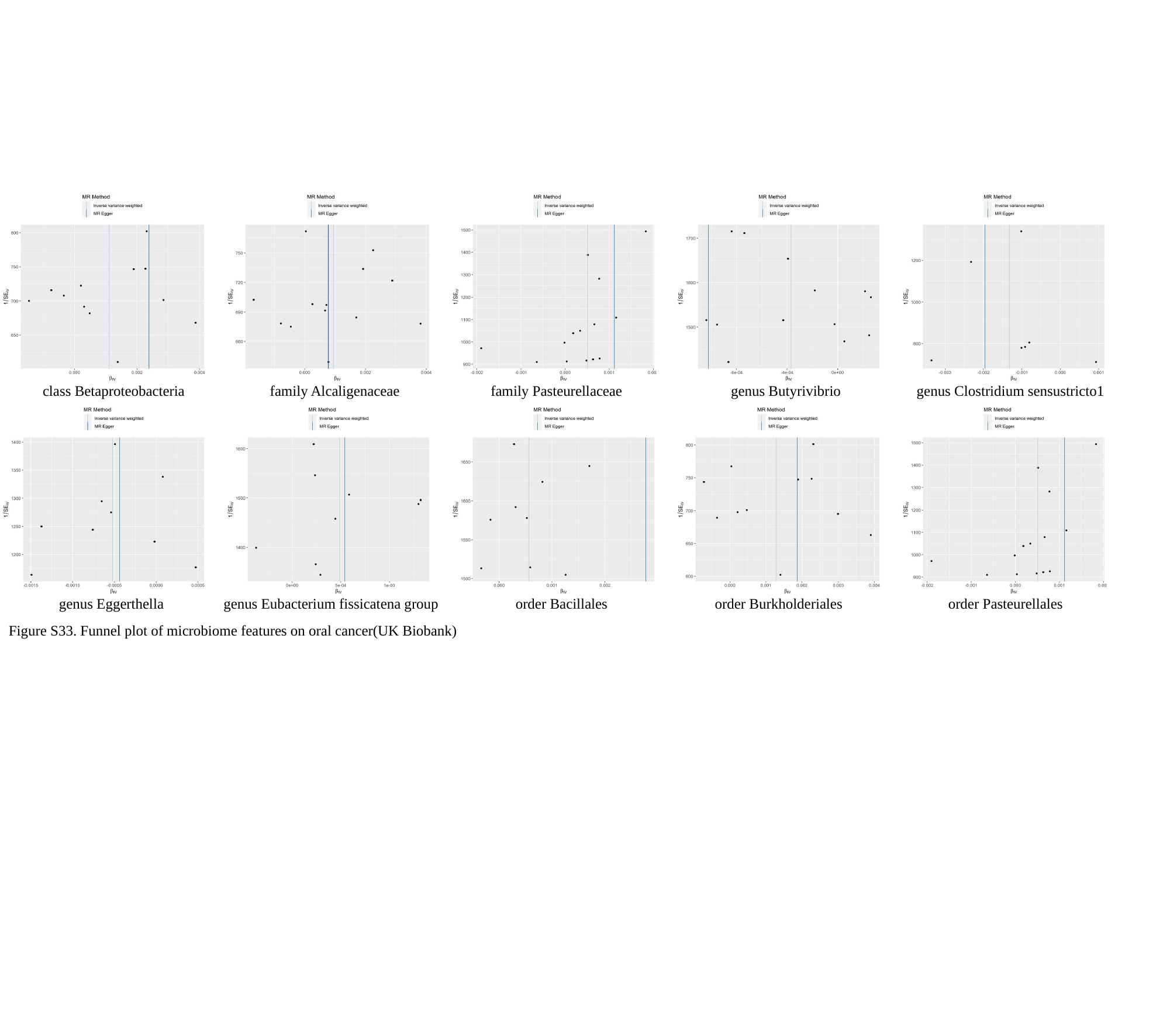

class Betaproteobacteria
family Alcaligenaceae
family Pasteurellaceae
genus Butyrivibrio
genus Clostridium sensustricto1
genus Eggerthella
genus Eubacterium fissicatena group
order Bacillales
order Burkholderiales
order Pasteurellales
Figure S33. Funnel plot of microbiome features on oral cancer(UK Biobank)

## Slide 34
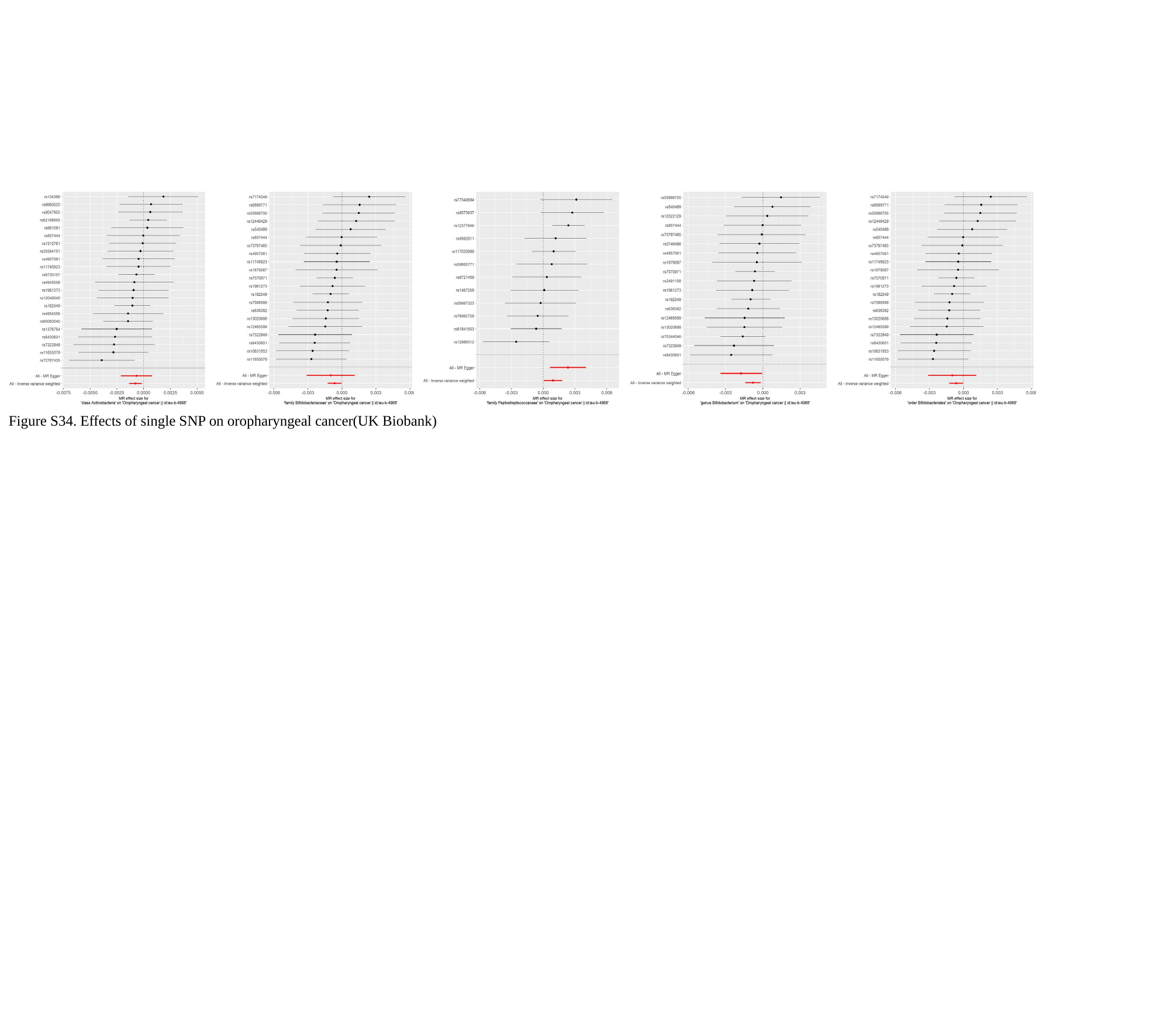

Figure S34. Effects of single SNP on oropharyngeal cancer(UK Biobank)

## Slide 35
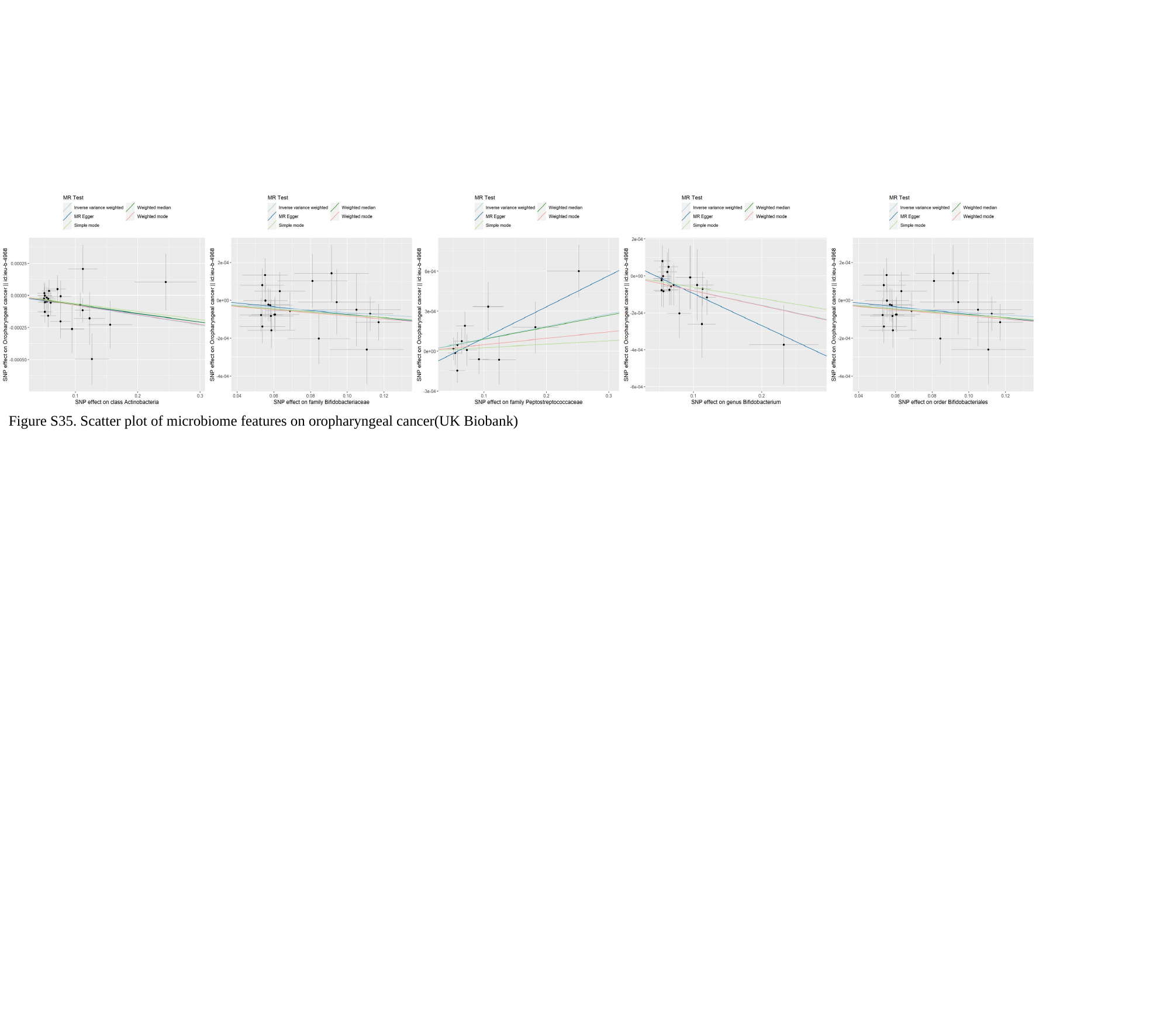

Figure S35. Scatter plot of microbiome features on oropharyngeal cancer(UK Biobank)

## Slide 36
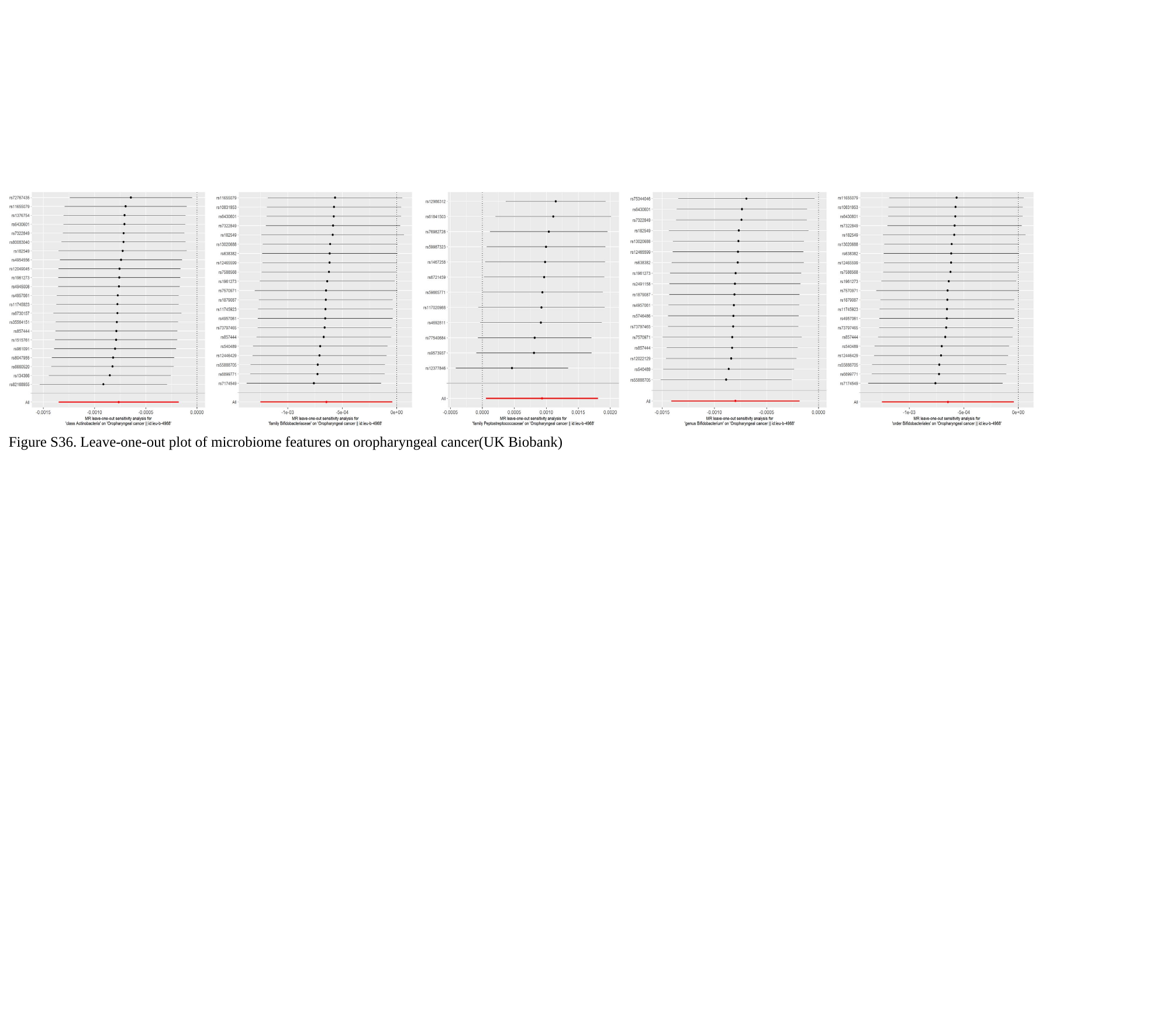

Figure S36. Leave-one-out plot of microbiome features on oropharyngeal cancer(UK Biobank)

## Slide 37
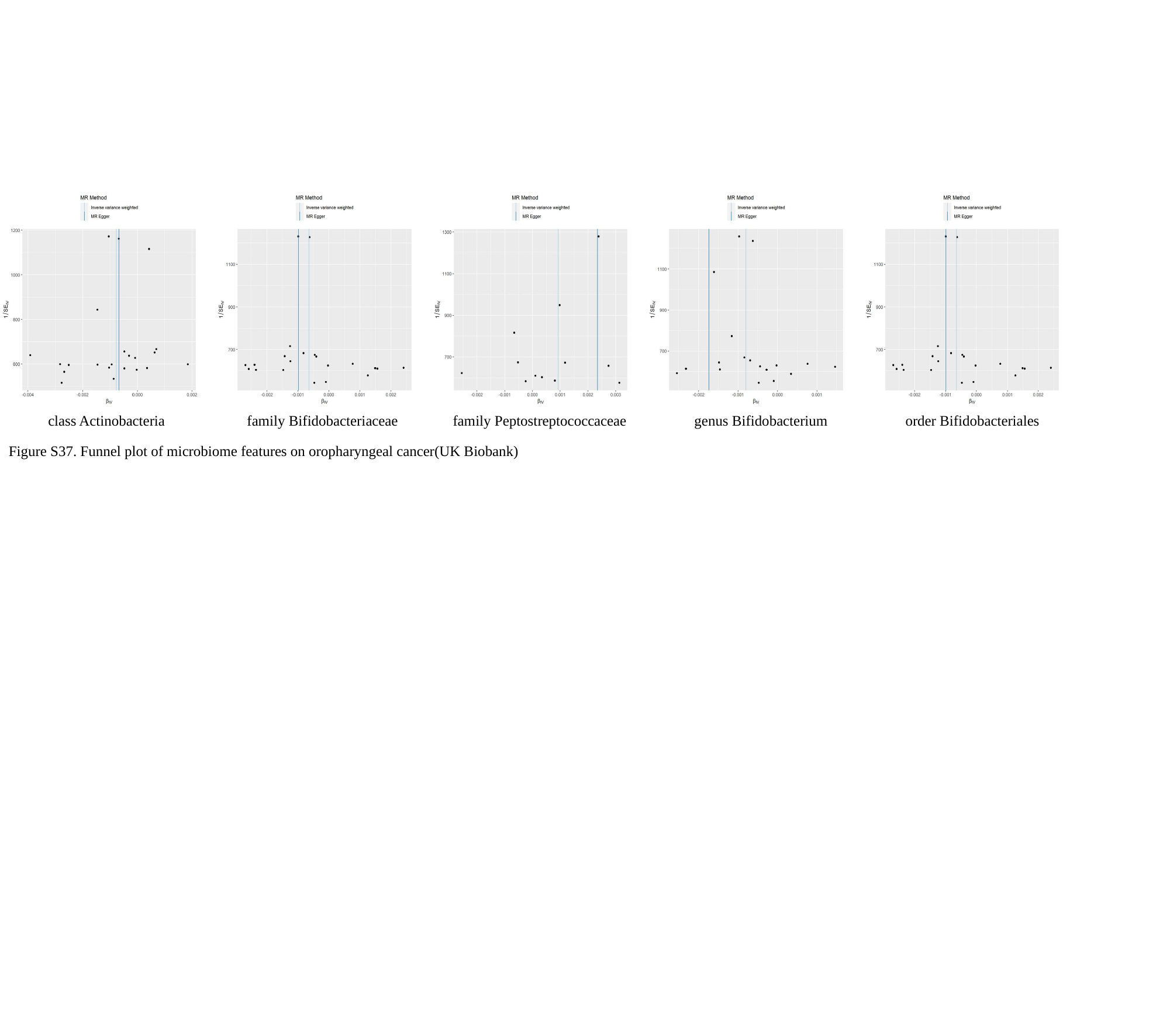

class Actinobacteria
family Bifidobacteriaceae
family Peptostreptococcaceae
genus Bifidobacterium
order Bifidobacteriales
Figure S37. Funnel plot of microbiome features on oropharyngeal cancer(UK Biobank)
